# Prediction of the space group and cell volume by training a convolutional neural network with primitive ‘ideal’ diffraction profiles and its application to ‘real’ experimental data

**DOI:** 10.1107/S1600576725002419

**Published:** 2025-04-25

**Authors:** Hiroyuki Ozaki, Naoya Ishida, Tetsu Kiyobayashi

**Affiliations:** a Research Institute of Electrochemical Energy, Department of Energy and Environment National Institute of Advanced Industrial Science and Technology (AIST) 1-8-31 Midorigaoka Ikeda Osaka563-8577 Japan; Australian Synchrotron, ANSTO, Australia

**Keywords:** space group classification, deep learning, powder X-ray diffraction, ensemble averaging, multi-task learning

## Abstract

This study describes a deep learning approach to predict the space group and unit-cell volume of inorganic crystals from their powder X-ray diffraction profiles. Using an inorganic crystallographic database, convolutional neural network (CNN) models were successfully constructed with the δ-function-like ‘ideal’ X-ray diffraction profiles derived solely from the intrinsic properties of the crystal structure, which are dependent on neither the incident X-ray wavelength nor the line shape of the profiles. We examined how the statistical metrics (*e.g.* the prediction accuracy, precision and recall) are influenced by the ensemble averaging technique and the multi-task learning approach; six CNN models were created from an identical data set for the former, and the space group classification was coupled with the unit-cell volume prediction in a CNN architecture for the latter. The CNN models trained in the ‘ideal’ world were tested with ‘real’ X-ray profiles for eleven materials such as TiO_2_, LiNiO_2_ and LiMnO_2_. While the models mostly fared well in the ‘real’ world, the cases at odds were scrutinized to elucidate the causes of the mismatch. Specifically for Li_2_MnO_3_, detailed crystallographic considerations revealed that the mismatch can stem from the state of the specific material and/or from the quality of the experimental data, and not from the CNN models. The present study demonstrates that we can obviate the need for emulating experimental diffraction profiles in training CNN models to elicit structural information, thereby focusing efforts on further improvements.

## Abstract 2

This study describes a deep learning approach to predict the space group and unit-cell volume of inorganic crystals from their powder X-ray diffraction profiles.

## 1. Introduction

For experimentalists who are dealing firsthand with newly synthesized materials, powder X-ray diffraction (XRD) is a convenient and indispensable technique for their characterization. Reconstruction of the atomic and molecular arrangement in three-dimensional space from the XRD profile, which is a one-dimensional vector caused by a scattering phenomenon, is a typical ill-posed inverse problem, that is, a unique solution is not readily attainable from the XRD profile, where the three-dimensional information is contracted merely into the intensity as a function of the diffraction angle. Formidable experience and expertise are required just for the initial setup of the crystal structure model, which in turn would be subject to further refinements such as Rietveld analysis.

The advent of deep learning technology, a type of machine learning that requires no explicit descriptor, is now shaping modern society more rapidly than ever imagined, including efforts such as AlphaGo (Silver *et al.*, 2016 ▸), GPT-3 (Floridi & Chiriatti, 2020 ▸) and Stable Diffusion (Rombach *et al.*, 2022 ▸). Materials science is no exception to this trend, though somewhat belatedly (Park *et al.*, 2017 ▸; Vecsei *et al.*, 2019 ▸; Liang *et al.*, 2020 ▸; Oviedo *et al.*, 2019 ▸; Ziletti *et al.*, 2018 ▸; Xie & Grossman, 2018 ▸; Liu *et al.*, 2019 ▸; Lolla *et al.*, 2022 ▸). Unlike the conventional Hanawalt method or others based on pattern matching, an advantage of applying deep learning technology to crystallography is that it can deal with any materials that have hitherto unknown structures. Previous studies have demonstrated that deep neural networks can assign the space group to which an inorganic compound belongs and predict its unit-cell parameters from one-dimensional XRD profiles (Park *et al.*, 2017 ▸; Vecsei *et al.*, 2019 ▸; Oviedo *et al.*, 2019 ▸) or two-dimensional diffraction images (Ziletti *et al.*, 2018 ▸). In these studies, the deep neural networks were trained by simulated profiles derived from the crystal structures in in­organic materials databases. For the networks to be applicable to experimental data, training and validation profiles were calculated by emulating real diffraction profiles as closely as possible, for example, simulating the line shape using a Voigt function onto which noise and fluctuations were overlaid.

The present study has two purposes. The first is to demonstrate the possibility of dividing the problem of eliciting structural information from an XRD profile into two schemes:

Scheme 1: Training convolutional neural network (CNN) models through a supervised learning process with only an *intrinsic* property of the material itself – the crystal structure factor *F*_*hkl*_ [see equation (6)].

Scheme 2: Extracting adequate signals from the experimental XRD profiles and feeding them into the CNN models after a proper conversion.

We have noticed that these schemes can be tackled independently and hence can be developed in separate computational systems. The present study mainly focuses on Scheme 1. The strategies based on these schemes are contrary to previous studies, wherein the models were trained with XRD profiles that simulate the experimental data. As the structure factor is independent of the experimental conditions (*e.g.* the incident X-ray wavelength, line shape and noise), the models in this study are trained, as it were, in an ‘ideal world’ (Section 3.3). The CNN models trained in the ideal world were then tested in the ‘real world’ using the experimental XRD profiles of eleven inorganic materials (Section 4.2). Since Scheme 2 is not covered in this study, the peaks were manually extracted from experimental profiles. The implications of these two separate schemes are addressed in the *Conclusions*[Sec sec5].

The second purpose of this study is to examine the effect of the ensemble averaging technique (Hansen & Salamon, 1990 ▸; Breiman, 1996 ▸; Lakshminarayanan *et al.*, 2017 ▸) and the multi-task learning (MTL) approach (Sener & Koltun, 2018 ▸) in improving the generalization performance. Concerning ensemble averaging, six CNN models were prepared from a single training data set by changing the randomizing process. In addition to the space group classification, the CNN models were trained through an approach that simultaneously predicts the unit-cell volumes. The reproducibility was confirmed with five training data sets.

As the crystallographic database, we opted for PowCod (Altomare *et al.*, 2015 ▸), which has *ca* 80000 entries of experimentally observed inorganic structures. Although several crystallographic databases have more entries than PowCod, one has to be careful in choosing a database because many forbid using their data for machine learning.

When tested in the real world with the eleven inorganic materials, the CNN models trained in the ideal world (Scheme 1) fared well, despite the primitive method of extracting signals from the experimental profiles (Scheme 2). The cases where a mismatch occurred were scrutinized material by material to identify the cause of the problem.

## 2. Data preparation

The data sets that were used in this study are sets of simulated diffraction profiles of inorganic crystals, each of which is labelled with the space group and primitive cell volume. The profiles and labels were calculated and analysed from structural data in the crystallographic database. Our CNN models were trained and validated with the profiles as the input and the labels, space groups and primitive cell volumes as the supervised data (*i.e.* the correct answer).

### 2.1. Crystallographic information files for data sets

PowCod is a freely available crystallographic database of inorganic crystals provided by the Institute of Crystallography based in Bari, Italy, derived from the Crystallography Open Database (COD) (Gražulis *et al.*, 2009 ▸), which encompasses organic, inorganic and metal–organic compounds. As mentioned in the *Introduction*[Sec sec1], we used PowCod despite its smaller number of entries than other commercially available databases because their contracts forbid their use for machine learning. Among the 81120 entries of inorganic crystals in PowCod, we successfully obtained 74882 crystallographic information files (CIFs) that can be read by the library *pymatgen* (Ong *et al.*, 2013 ▸), which allows for a virtual atom consisting of two or more elements or vacancies according to their fractional occupancies on a site. Because nearly half of the CIFs in the database involve virtual atoms, additional care was taken when using libraries that did not support virtual atoms, such as *Atomic Simulation Environment* (*ASE*) (Larsen *et al.*, 2017 ▸).

### 2.2. Analysing space groups

The CIFs may contain information about the space group to which the crystal structure belongs, as the Hermann–Mauguin notation in _space_group_name_H-M_alt and the space group number in _space_group_IT_number or _symmetry_Int_Tables_number. However, as we found that some of the structures are mistakenly classified into a lower symmetry group (*i.e.* a subgroup), we analysed all the space groups in the CIFs using the library *Spglib* (Togo *et al.*, 2018 ▸). As a result, 2225 structures (*ca* 3% of the total) were relabelled.

To secure a sufficient number of items in each space group, we limited the classifying space groups to the 57 frequently occurring groups listed in Table 1 ▸, totalling 62830 items, referred to as data set *D*(∞).

### 2.3. Distribution of the primitive cell volume and the atomic number density

We examined the effect of excluding ‘outliers’ from the data sets with respect to the unit-cell volume and the atomic number density, both of which obey a log-normal distribution, as described below (the former is also used as supervised data for the CNN with the MTL approach).

While the diffraction profile and the space group are independent of the choice of unit cell, the cell volume is not. We opted for the primitive cell over the conventional cell to guarantee the uniqueness of the supervised data for the MTL approach. We first constructed the conventional cell from the CIFs using *Spglib*, from which the primitive cell with the minimum cell volume was in turn extracted using the algorithm proposed by Setyawan & Curtarolo (2010 ▸). Our implementation was validated by the agreement between the simulated diffraction profiles generated from the conventional and primitive cells.

**Figure 1:**
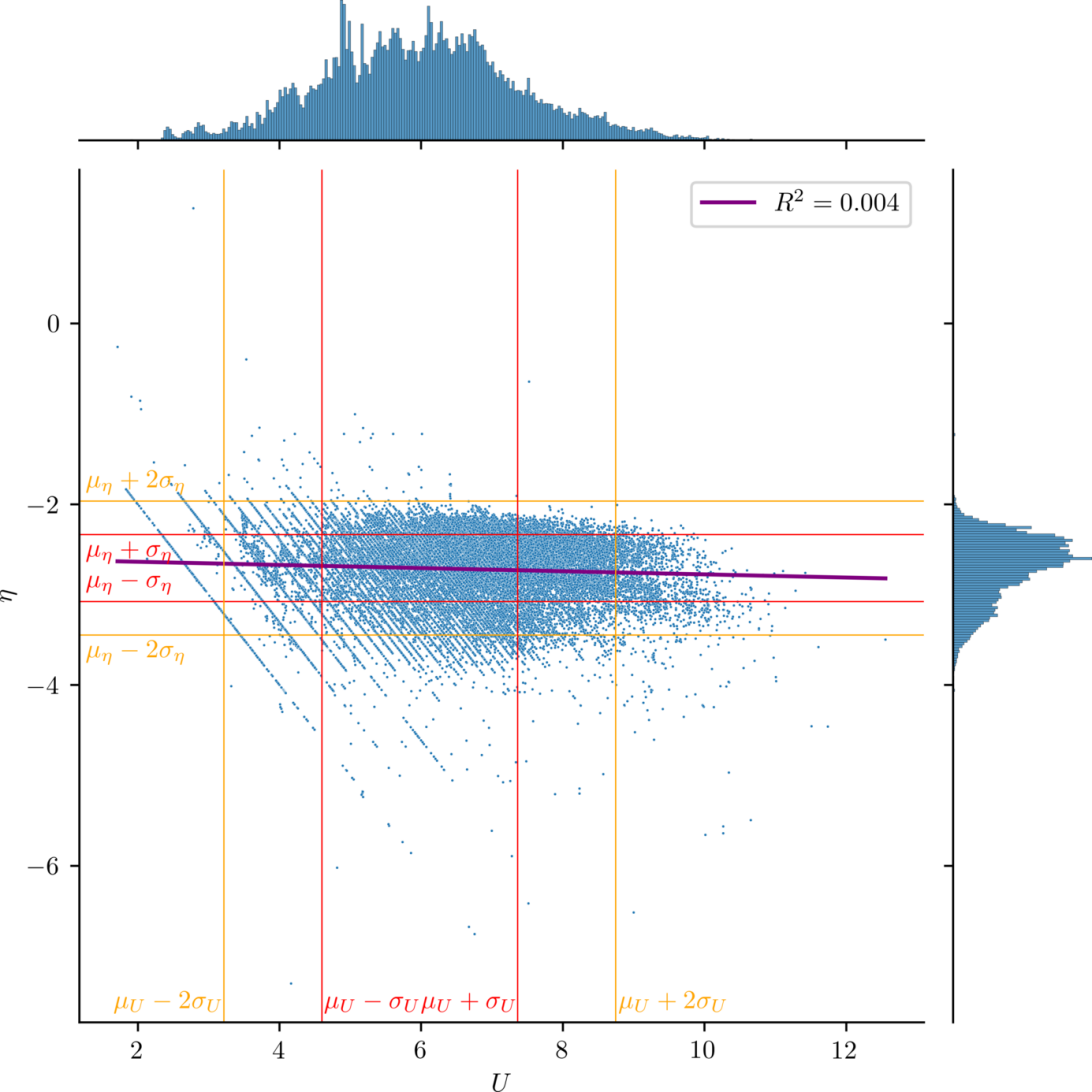
Distribution of the logarithmic primitive cell volumes, *U* in equation (1), and the logarithmic atomic number density, η in equation (2).

Fig. 1 ▸ shows the distribution of the logarithmic primitive cell volume *U* and the logarithmic atomic number density η of the 62830 structures belonging to the 57 space groups in Table 1 ▸: 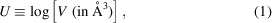

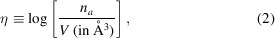
where *V* and *n*_*a*_ are the primitive cell volume and the number of atoms in it, respectively. As the figure suggests that both *U* and η independently obey a normal distribution, one can examine the effect of excluding from the training data sets the outliers lying outside of ±σ and ±2σ, where σ is the standard deviation. Table 2 ▸ lists the number of entries for seven data sets after excluding outliers, where *D*(∞) is the data set without excluding the outliers, *D*(σ_*U*_) excludes from *D*(∞) the outliers outside of ±σ with respect to *U*, *D*(σ_*U*+η_) excludes those outside of ±σ with respect to both *U* and η, and so forth.

Standardization of the normal distribution, referred to as *Z*-score normalization, is often effective in improving the prediction accuracy, not only in the deep learning approach but also in the machine learning approach and other applications. We hence used the following quantity as the supervised data for the primitive cell volume prediction,
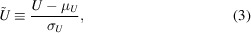
where μ_*U*_ and σ_*U*_ are the mean and standard deviation of *U*, respectively. By definition, the distribution of 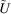 centres at 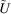 = 0 with a standard deviation of 1.0.

### 2.4. Training and validation data sets

We created five mutually disjointed validation data sets, 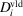 (1 ≤ *i* ≤ 5), under the following considerations. Our preliminary investigation suggested that the CNN models trained by the outlier-excluded data set predicted very poorly the space groups and cell volumes of the outliers, *i.e.* the CNN models are not good at extrapolating their knowledge. Hence, to extract purely the effect of outlier exclusion during training, we selected the validation data set from the narrowest range in Table 2 ▸, namely from *D*(σ_*U*+η_). From each of the 57 space groups in Table 1 ▸ in *D*(σ_*U*+η_), we selected 80 items and divided them into five sets, each of which has 16 items. In general, the parent data set is divided into training and validation data sets by maintaining similar distributions to evaluate the generalization performance; thus in the present study, the imbalanced distribution of the space groups in PowCod (the distribution of real materials) is reflected in both the training and validation data sets. In this study, however, an equal number of items were selected from each space group to focus on the statistical variation among the space groups. Each of the validation data sets, 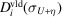 (1 ≤ *i* ≤ 5), hence has 57 × 16 = 912 items. As the validation data sets are extracted only from *D*(σ_*U*+η_), we abbreviate 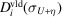 as 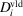. Note that the intersection of any two validation data sets is the empty set, *i.e.*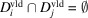 (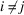). When selecting 80 items from each space group, we forestalled ‘training-data leakage’ by excluding from the validation data set any structures which belong to the identical space group and of which all the diffraction peaks fall within the five bins of the first convolution layer of the CNN (see Section 3.2) of another structure in the training data set, lest almost identical structures be included in both the training and validation data sets. The issue of duplicated entries (*i.e.* compounds having the same chemical formula being registered multiple times) is also prevented in this way if duplicated structures match the above criterion. If, on the other hand, the profiles of duplicated formulae differ and lie outside the criterion, we consider they should be treated as different materials because their unit-cell volumes do not coincide. (Note that the chemical formulae are irrelevant in our CNN models as they are used in neither the training nor the validation.)

From the seven data sets listed in Table 2 ▸, five training data sets 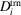 were created by subtracting five validation data sets 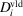 (1 ≤ *i* ≤ 5) as 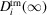 = 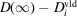, 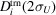 = 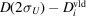,…, 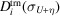 = 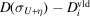. By changing the randomizing seeds, six CNN models, I–VI, were created from each training data set 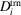 to assess the effect of the ensemble averaging technique (see Section 4.1.1). The training data sets 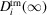 were also used to train the CNN models that simultaneously predict the space group and primitive cell volume. A total of 240 CNN models were created in this study: five training data sets (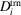) × (seven data set ranges + 1 for the MTL) × six models (I–VI).

### 2.5. Simulated diffraction profiles

Each of the simulated diffraction profiles consists of the diffraction intensity *I*_*hkl*_ as a function of the magnitude of the reciprocal-lattice vector, 1/*d* ≡ 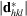, where *hkl* are the reflection indices of the crystal lattice:
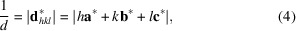
and 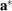, 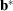 and 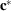 are the reciprocal basis vectors. The diffraction intensity *I*_*hkl*_ is the square of the crystal structure factor *F*_*hkl*_:
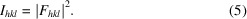
The structure factor is given by 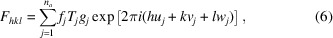
where *u*_*j*_, *v*_*j*_ and *w*_*j*_ are the fractional coordinates for each atom *j*, *f*_*j*_ is the atomic scattering factor, *T*_*j*_ is the Debye–Waller factor, and *g*_*j*_ is the partial occupancy. We adopted the following approximate expressions for *f*_*j*_ and *T*_*j*_:
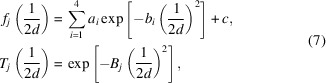
where the nine parameters *a*_*i*_, *b*_*i*_ and *c* were taken from Ibers & Hamilton (1974 ▸) and *B*_*j*_ = 2.0.

Neither 1/*d* nor *I*_*hkl*_ depends on the incident beam (*e.g.* its wavelength or Lorentz–polarization factor), nor is the intensity *I*_*hkl*_ influenced by the experimental conditions [*e.g.* the signal-to-noise ratio or the crystal size of the specimen]. The CNN models are hence trained and validated, as it were, in an ‘ideal’ world, such that the discrete δ-function-like profiles are derived from the inherent properties of the structure, free from factors such as line shape and noise.

The range 0.1131 < *d*^−1^ (Å^−1^) < 1.0625 was divided into 10001 bins. For each structure, *I*_*hkl*_ and 1/*d* were calculated for all the combinations of (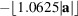, 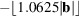, 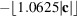) ≤ (*h*, *k*, *l*) ≤ (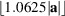, 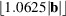, 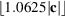) and each calculated *I*_*hkl*_ was accumulated on the corresponding 1/*d* bin. In this way, the extinction rule, multiplicity and accidental overlapping of the signals are automatically taken into account. An example of binning and accumulating *I*_*hkl*_ where two diffraction intensities from two distinct planes accidentally overlap is shown in Fig. S13 in the supporting information. Finally, the intensity was normalized in such a manner that the maximum signal intensity was 1.0 for each structure. If the incident X-ray beam is Cu *K*α excitation, these 1/*d* bins correspond to a diffraction angle 10° < 2θ < 110° with an interval of 0.01°. On the basis of the availability of the parameters in *International tables for X-ray crystallography* (Ibers & Hamilton, 1974 ▸), we limited the atomic number to less than 98 (Cf).

### 2.6. Preprocessing of experimental data

This section describes Scheme 2 mentioned in the *Introduction*[Sec sec1], *i.e.* extraction of the peak positions and intensities from experimental XRD profiles and their conversion to feed the CNN models trained using Scheme 1. The peak intensities *I*(2θ) extracted from an experimental XRD profile involve the Lorentz–polarization factor *f*_LP_ as a function of half of the diffraction angle θ, which depends on the wavelength λ of the incident X-rays. We hence have to transform *I*(2θ) into the intensity without the Lorentz–polarization factor, *I*_*hkl*_ in equation (5), as a function of the reciprocal interplanar spacing, 1/*d* in equation (4), to feed the CNN models constructed in this study. The diffraction angle 2θ is converted to 1/*d* through the Bragg condition, 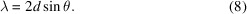
The experimentally observed diffraction intensity is proportional to *I*(2θ) given by
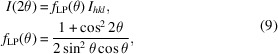
where *I*_*hkl*_ is given in equation (5).

The peak intensities and their positions were extracted from the experimental diffraction profiles using the find_ peak_cwt() function in the Python library *SciPy* (https://scipy.org/), which detects peaks on the basis of the wavelet transform (Du *et al.*, 2006 ▸). Because the detection is inevitably subject to errors, such as spike noise or a signal buried in the background, certain manual adjustments were occasionally required. The extracted peak intensities were corrected when the signals from multiple wavelengths (*e.g.K*α_1_ and *K*α_2_) were overlaid in the experimental diffraction profile. Our CNN models trained in the ‘ideal’ world in Section 2.5 were fed with the input data, after the extracted peak intensity had been re-scaled to *I*_*hkl*_ by dividing *I*(2θ) by *f*_LP_ [see equation (9)] and normalized so that the maximum signal intensity was 1.0. The influence of the noise, impurities and undetected signals is discussed on a case by case basis in Section 4.2.

## 3. Neural networks

### 3.1. Convolutional neural networks

This section describes the general aspects of the CNN. The architecture of the CNN is fundamentally a type of feed-forward neural network consisting of an input layer, intermediate layers and an output layer. Each layer is represented as a vector of perceptrons modelling neurons, and the layers are connected by weights. The output *y*_*i*_ of a layer *i* is expressed in terms of the input *x*_*i*_ to the layer *i*, the weight *W*_*i*_ between the layers *i* and *i* − 1, and the bias *b*_*i*_ through a nonlinear function *f* called the activation function as follows: 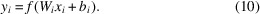
A layer in which all perceptrons are connected is called fully connected, while a locally connected layer is called a convolutional layer. Along with the convolutional layer, a pooling layer is commonly used that outputs the maximum or average of the inputs. A dropout layer is occasionally used to suppress overfitting, in which an excessive adaptation to the training data set reduces the prediction accuracy for unknown data, *i.e.* generalization performance.

The model instantiating the CNN architecture, referred to as the CNN model, is iteratively trained by changing its weights and biases to minimize the error between the supervised data and the output. The supervised data are essential for training the model and must be prepared in advance. The error is evaluated by a loss function, the choice of which depends on the problem under consideration. For example, the cross-entropy error, which measures the difference between two probability distributions, is often used as the loss function for classification problems. The mean squared error, which measures the average squared difference between numerical values, is widely used for regression problems. In training a CNN model, gradient methods, also known as optimization functions, are used to compute the weights and biases to minimize the error because they are computed by differentiating the loss function. Among the various optimization functions, stochastic gradient descent (SGD) and adaptive moment estimation (ADAM) are widely used.

The training data set is divided into non-recoverable sets, referred to as mini-batches, with which CNN models are iteratively trained. Typically, the mini-batch size is a constant that must be set prior to training. These preset parameters are called hyperparameters to distinguish them from the parameters of the neural networks. An appropriate mini-batch size leads to a higher training speed and prediction accuracy. One ‘epoch’ elapses when all the mini-batches have been input to the training session. The entire training session is completed after several thousand epochs.

### 3.2. CNNs for space group classification

**Figure 2:**
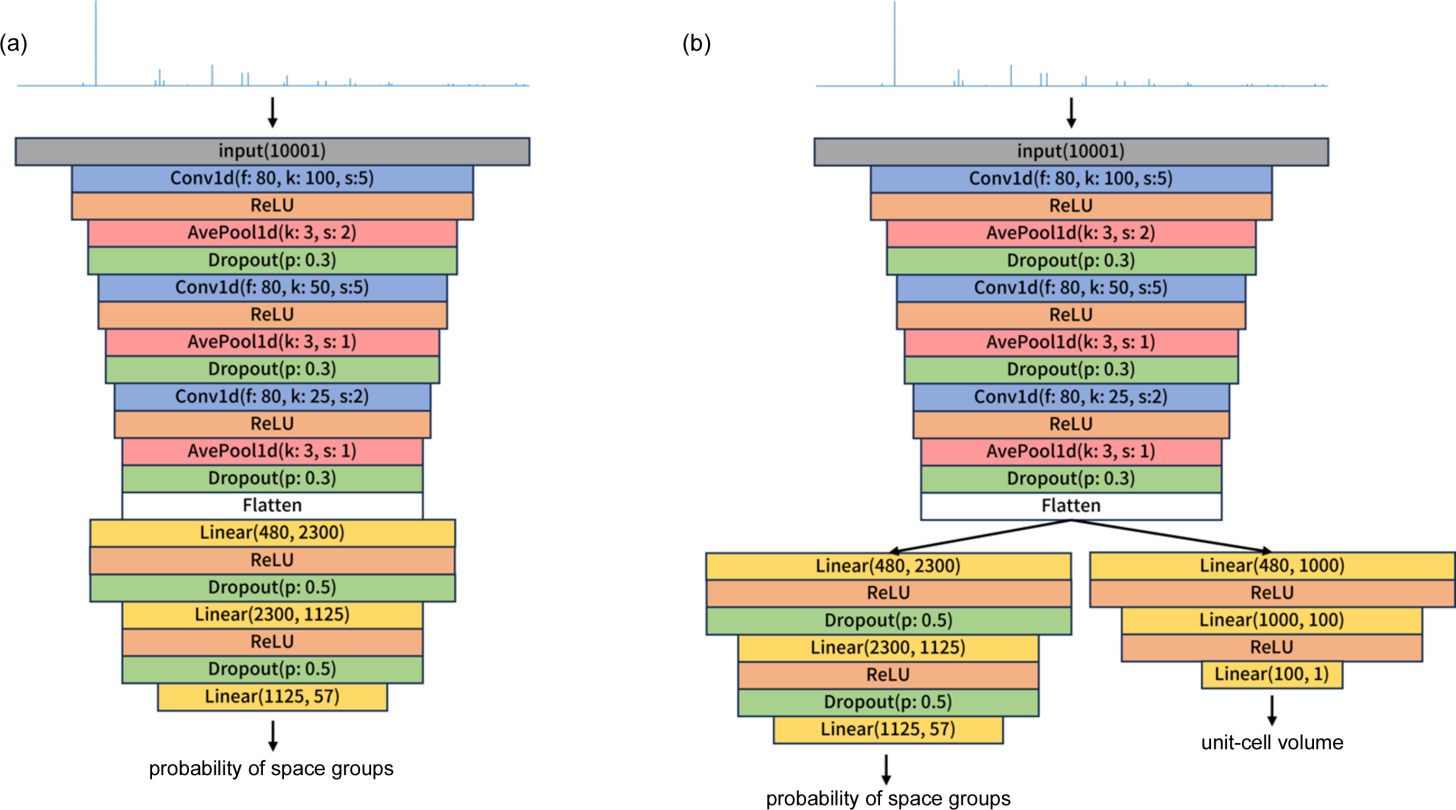
CNNs for (*a*) classifying only the space group (SpgNet) and (*b*) predicting the space group and cell volume using a multi-task learning approach (SpgVolNet) from XRD profiles. The diffraction intensity *I_hkl_* as a function of the reciprocal interplanar distance 1/*d* divided into 10001 bins is loaded to the input layer, depicted in grey. The convolutional layers are shown in blue, with *f* channels of kernel size *k* and stride *s*. The activation functions are shown in orange. The dropout layers (in green) are inserted to control overfitting in (*a*). The average pooling layers are shown in red with kernel size *k* and stride *s*. The layer in white converts its input to one dimension without changing the values. The layers in yellow are fully connected. In the output layers in both (*a*) and (*b*), the identity function is adopted as the activation function.

In this study, two CNNs were developed (Fig. 2 ▸) following the architecture proposed by Park *et al.* (2017 ▸). We did not attempt to optimize the architecture because we focused our attention on the feasibility of training CNN models with δ-function-like profiles, as well as on the effect of ensemble averaging and an MTL approach. There is room for optimization and improvement by means of, for example, neural architecture search (Zoph & Le, 2017 ▸). Fig. 2 ▸(*a*) depicts the CNN architecture to classify only the space group (the single-task learning approach), referred to as SpgNet. The architecture depicted in Fig. 2 ▸(*b*) simultaneously predicts the space group and the primitive cell volume using an MTL approach, referred to as SpgVolNet.

In Fig. 2 ▸(*a*), *i.e.* SpgNet, the input layer (in grey) receives the diffraction intensities *I*_*hkl*_ as a function of 1/*d*, divided into 10001 discrete bins, as described in Section 2.5. The intermediate layers consist of three convolutional blocks and two fully connected blocks. The last convolutional block and first fully connected layer are connected by a flattening layer represented in white. This flattening layer transforms the feature maps into a one-dimensional vector suitable for input to the fully connected layers. Each convolutional block consists of a convolutional layer (shown in blue), an activation function (in orange) and a pooling layer (in red). Each fully connected block has a fully connected layer (in yellow) and an activation function (in orange). Both types of block have a dropout layer (shown in green) to control overfitting. The number of perceptrons in the fully connected layers is determined by that in the output layer. We adopted the rectified linear unit (ReLU; Nair & Hinton, 2010 ▸) as the activation function in the intermediate layers. The output layer has 57 perceptrons to register the probability of the input, and these are classified into the 57 space groups considered in this study (see Section 2.2). The architecture is very faithful to the general CNN architecture.

MTL is a technique aimed at improving the learning efficiency and generalization performance of each task by learning multiple related tasks simultaneously (Sener & Koltun, 2018 ▸). In this context, a task refers to a problem that specifies desired outputs or objectives for a given input and aims to solve it by minimizing the error between those outputs and the predicted results. The fundamental idea behind this approach is that, by incorporating common layers to share information and features across tasks, the model can acquire more knowledge than when learning each task independently. When learning individual tasks separately, there may be insufficient training data or a risk of overfitting to specific tasks. However, in MTL, knowledge is shared across different tasks to enhance generalization performance and help prevent overfitting. Specifically, it is common to adopt a structure in which certain layers of the neural network are shared among tasks, while each task has its own dedicated output layer. The features learned in this shared part can capture important patterns that are common across multiple tasks, complementing the task-specific parts. For example, when performing image classification (object recognition) and object detection (object localization) simultaneously, using shared information about the shape and edges of objects can improve the prediction accuracy of both tasks. In this context, the synergistic effects between tasks can lead to better performance than learning each task individually.

In this study, we developed a CNN with a task to classify space groups and a task to predict the cell volume of primitive cells, as shown in Fig. 2 ▸(*b*), referred to as SpgVolNet. The counterpart CNN shown in Fig. 2 ▸(*a*) that predicts only the space groups is referred to as SpgNet. From the input layer to the flattening layer of SpgVolNet is the shared part, having the same structure as SpgNet, and this is followed by fully connected blocks specific to each task. The task specific to space group classification has the same hierarchical structure as SpgNet, but only the space group classification task retains this identical architecture. In contrast, the task specific to cell volume prediction consists of two fully connected blocks, without a dropout layer, and an output layer. The output layer has one perceptron to register a numerical value of the predicted cell volume. The identity function was adopted as the activation function in both output layers, to adapt to the loss functions described in the next section.

### 3.3. Training

For classification tasks, the cross-entropy error is widely used to measure the difference between two probability distributions. There are two main variants frequently employed in neural networks: binary cross-entropy (BCE) and softmax cross-entropy (SCE). The former is generally suitable for multi-label classification, where each label can be treated as an independent Bernoulli random variable, while the latter is suitable for single-label multi-class classification, where exactly one class out of *C* possible classes is correct. Because our problem requires assigning a unique space group to each input (see Section 2.2), we adopted SCE rather than BCE. For space group classification in both SpgNet and SpgVolNet, we used the SCE error as the loss function.

The softmax function converts the logits {*z*(*i*, *j*)} into predicted probabilities,
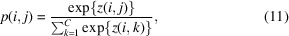
and the SCE error 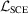 is defined as 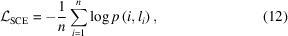
where *C* (= 57 in this study) is the number of classes, *p*(*i*, *j*) is the predicted probability that sample *i* belongs to class *j*, and *l*_*i*_ is the true class label (*i.e.* the correct space group). In practice, nn.CrossEntropyLoss in *PyTorch* implements this by the log–sum–exp trick using the logits {*z*(*i*, *j*)} internally, but the result is equivalent to the above probability-based definition.

For the cell volume prediction in SpgVolNet, the mean absolute error (MAE) was adopted as the loss function, as the MAE predicts slightly more accurately than the mean squared error in our preliminary experiments. This loss function is defined as
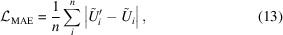
where 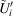 is the predicted primitive cell volume of structure *i* and 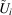 is its supervised value.

MTL has distinctive features in terms of loss functions. Typically, an individual loss function 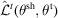 is defined for each task *t* and the overall loss 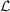 is computed as a weighted combination of these losses using weights *c*^*t*^:
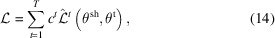
where θ^sh^ represents the shared parameters and θ^t^ represents the task-specific parameters. By balancing multiple tasks in this way, the training of CNN models can effectively proceed using optimization functions. Note that, while the above formulation distinguishes between shared parameters θ^sh^ and task-specific parameters θ^t^, our implementation does not explicitly separate θ^sh^. Instead, each task (*i.e.* space group classification and cell volume prediction) has its own dedicated set of parameters and loss functions, namely the SCE error 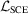 for classification and the mean absolute error 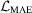 for regression. The overall MTL objective is then given by the weighted sum of 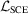 and 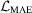.

One can adopt any appropriate optimization function for the MTL approach. This allows training to proceed in the same manner as other deep learning models, taking advantage of existing optimization algorithms while benefiting from the effects of the MTL approach. The MTL approach thus offers high flexibility in terms of loss functions and optimization, making it applicable to a wide range of problems. In this study, owing to its faster training and convergence time compared with SGD, the ADAM function was used to optimize the space group classification and cell volume prediction with the MTL approach. The ADAM function has three preset hyper­parameters: learning rate γ and learning coefficients β_1_ and β_2_. The default values were set as γ = 0.001, β_1_ = 0.9 and β_2_ = 0.999 for both trainings. As in the previous study by Park *et al.* (2017 ▸), the CNN model was trained through 5000 epochs.

Training CNN models with a random order in the data set usually improves the prediction accuracy more than training with a fixed order. Hence in our study, the training order of the data set was shuffled, which resulted in a variation in the prediction accuracy of every trained CNN model, depending on the randomizing process, even with the training data set 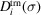 for a given pair of *i* and σ described in Section 2.4. By varying randomizations, we trained six independent CNN models, labelled I–VI, from each validation data set 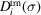. Each of the six models returns the probability 0 ≤ *p*_*m*_(*g*) ≤ 1 (*m* = I–VI) that the crystal structure would belong to a space group *g* ∈ *G*, where *G* is a set of the space groups listed in Table 1 ▸. All training experiments were carried out on our GPU server, which consists of 16 cores of CPU (Intel Xeon Processor Gold 6326), 512 GB memory and three GPUs (Nvidia A100 80 GB PCIe), although we have confirmed that a PC consisting of 24 cores of CPU (Intel Core i9-13900K), 128 GB memory and one GPU (Nvidia GeForce RTX 4090) is also viable for the experiments. Each training session for an SpgVolNet model took *ca* 6.5 h for the largest data set, *D*(∞), and *ca* 3 h for the smallest one, *D*(σ_*U*+η_), using an Nvidia A100 GPU. About 10 ms were required for an SpgVolNet model to return the inference after the input of XRD peak positions and intensities.

The prediction results are presented as a table of the space groups (as in Tables SI–SIX) in descending order of *p*_*m*_(*g*) and the ensemble mean probability given by 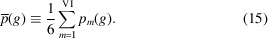


### 3.4. Statistical metrics

Space group classification is a multi-class classification problem, which determines to which of the multiple classes (space groups) an input belongs. The performance of multi-class classification is generally evaluated using statistical metrics such as prediction accuracy, precision, recall and *F*-score (Corriero *et al.*, 2023 ▸; Venkatraman & Carvalho, 2022 ▸; Liu *et al.*, 2019 ▸). Prediction accuracy is the proportion of correctly classified samples among all predictions, precision measures the proportion of correct predictions for a specific class, recall indicates the proportion of actual samples of that class correctly identified, and the *F*-score is the harmonic mean of precision and recall, representing a balance between the two. These metrics can be derived by examining the counts of true positives, false positives and false negatives recorded in a confusion matrix (Stehman, 1997 ▸), which is a table that aggregates the combinations of true and predicted classes. The confusion matrix enables gaining a detailed understanding of where the classifier performs well and where it makes mistakes.

Let **M** be a *C* × *C* confusion matrix, where *C* is the number of classes; *C* = 57 in this study. The element *M*_*ij*_ totals the number of items whose true label is *i* but which were classified as *j*, *i.e.* the diagonal element *M*_*ii*_ corresponds to correctly classified items. In the multi-class classification, the precision 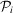 and the recall 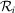 associated with the label *i* are defined using **M** by 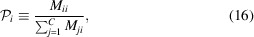

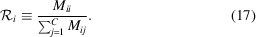
The precision 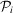 is the rate of items correctly classified to be *i*, *M*_*ii*_, among the total items classified to be *i*, 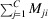, *i.e.* the higher the precision 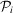, the less likely that items belonging to *j* (≠ *i*) are wrongly classified to be *i*, or the lower the likelihood of false positive cases for *i*. The recall 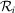 is the rate of correctly classified items among the total items whose true label is *i*, 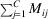, which is the total number of items in the validation data labelled *i* and is 16 for all *i*s in the five validation data sets 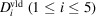 described in Section 2.4, *i.e.* the higher the recall 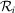, the less likely it is that items belonging to *i* are wrongly classified to be *j* (≠ *i*) or the lower the likelihood of false negative cases for *i*. The macro means of these metrics are 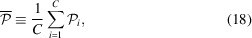

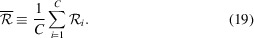
Precision and recall are often in a trade-off relationship, so their harmonic mean, termed the *F*-score, is occasionally invoked in evaluating classification models:
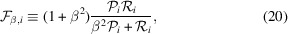
where β is an arbitrary multiplier that weights the precision over the recall. The (overall) prediction accuracy is defined by 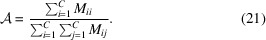
Because 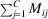 = 16 regardless of *i* in this study, 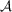 = 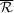. An example of a confusion matrix is provided in the supporting information.

One confusion matrix, **M**, is derived from the probability *p*_*m*_(*g*) output by each of the six CNN models, *m* = I–VI, and their ensemble mean, 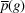 in equation (15). The element *M*_*ij*_ counts the structure belonging to space group *j* for which the highest probability was given to *p*_*m*_(*i*), yielding the confusion matrix **M**_*m*_, or 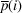 yielding 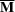. Hence, we can examine the effect of the ensemble averaging by comparing the following two means of a metric 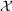 [*e.g.*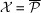 in equation (18)]. One is 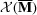 derived from 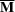 and the other is 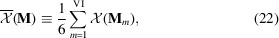
where 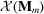 is the metric derived from confusion matrix **M**_*m*_. We refer to 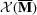 as the ‘ensemble mean’ and 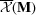 as the ‘specific mean’.

## 4. Results and discussion

### 4.1. Evaluation of the trained CNN models

#### 4.1.1. Space group classification

As described in Section 2.3, the primitive cell volume *V* and the number density of atoms *n*_*a*_/*V* obey a log-normal distribution. Table SXI in the supporting information summarizes the effect of excluding from the training data set the outliers lying outside of ±2σ or ±σ (where σ is the standard deviation) of the log-normal distribution with respect to 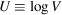 and/or 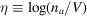. The SpgNet models trained by the widest range, *D*(∞), fare best in classifying the space group of items in 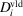 (1 ≤ *i* ≤ 5), despite the fact that the items in these validation data sets were selected from the narrowest data set *D*(σ_*U*+η_). Learning the outliers reinforces the SpgNet models. Conversely, the SpgNet models trained by narrow data sets perform poorly in classifying the space group of items lying outside the data sets by which they were trained (Hendrycks & Gimpel, 2017 ▸; Ovadia *et al.*, 2019 ▸).

The metric 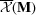 where 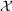 = 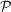, 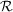, 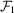 is the specific mean defined in equation (22) and 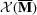 is the ensemble mean derived from the confusion matrix 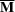 based on the ensemble mean probability 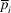 defined in equation (15). Mean results based on the five validation data sets 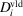 (1 ≤ *i* ≤ 5) are discussed. In the present study, the accuracy 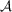 coincides with 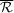 because of the fixed number of entries (16) in the validation data sets for each space group.

Table 3 ▸ examines the effect of the ensemble averaging technique and the MTL approach. Evidently, 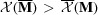 in terms of both the precision 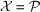 and the recall 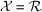 (which coincides with the accuracy 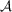 in this study), and consequently 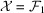. In particular, the improvement by ensemble averaging is significant for the precision 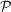, where the 95%-confidence zones do not overlap between 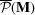 and 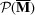, *i.e.* the ensemble averaging suppresses false positive cases. The results confirm that ensemble averaging, which is considered effective in multi-class classification problems (Hansen & Salamon, 1990 ▸; Breiman, 1996 ▸; Lakshminarayanan *et al.*, 2017 ▸), is also effective in space group classification. In contrast, for MTL, while its effect is not significant for the precision 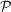, it improves the recall 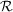, suggesting that MTL is effective in reducing false negative cases. The present study demonstrates that combining ensemble averaging and MTL complementarily improves the precision and recall.

#### 4.1.2. Cell volume prediction

**Figure 3:**
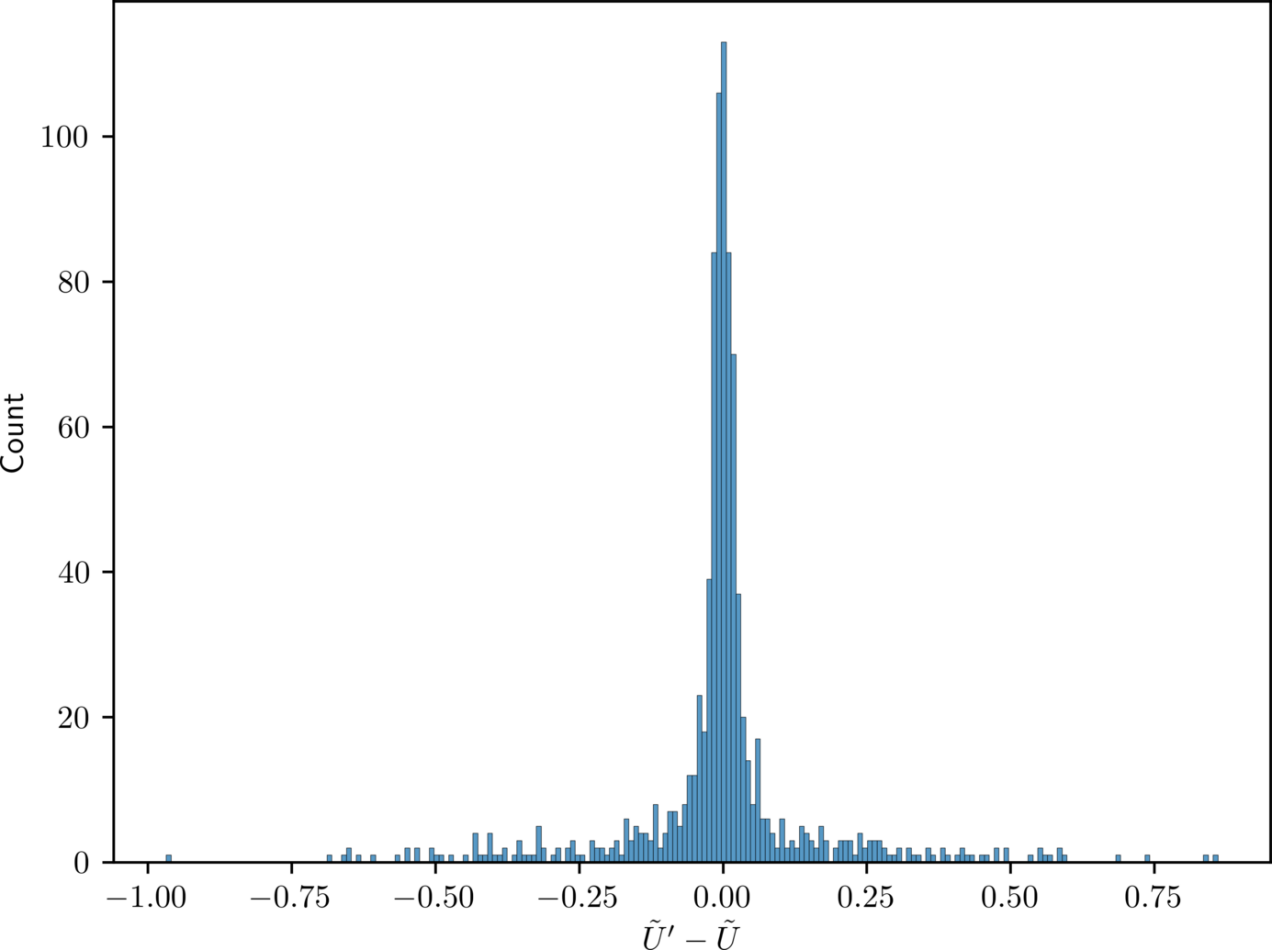
Distribution of the error in the standardized log-normal primitive cell volume, 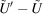 [equation (3)].

Fig. 3 ▸ shows the distribution in the error 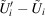, where 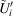 and 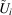 are, respectively, the predicted and supervised standardized logarithmic cell volumes of the structure *i* on 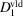 [see equation (3)]. The histogram obeys the Cauchy distribution, characterized by its wide tails, and not the normal distribution represented by the Gaussian function.

Stochastic phenomena obeying the Cauchy distribution are evaluated in terms of the median and quartiles, not of the mean and variance, which are indefinable for the Cauchy distribution. The statistical analysis of the prediction results suggests that 50% of the predicted cell volumes *V*′ lying between the 75% and 25% quartiles are in the range 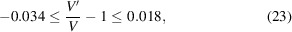
where *V* is the supervised (correct) cell volume. That is, the predicted primitive cell volume of 50% of the structures in the validation data sets deviates between 1.8% and −3.4% of the correct value.

### 4.2. Application to real experimental diffraction profiles

#### 4.2.1. Outline of the test results of the eleven inorganic materials

The SpgVolNet models to classify the space groups and predict the primitive cell volumes, trained in the ideal world (*i.e.* by the δ-function-like diffraction intensities *I*_*hkl*_), were tested with real experimental diffraction profiles (*i.e.* by a set of peak intensities extracted from them). The results are summarized in the supporting information. Among the eleven inorganic materials whose conceivable correct space group is included in Table 1 ▸, eight of them were unequivocally classified to the correct space group, for which all the SpgVolNet models, I–VI, assigned exactly 100% or close probability; they are TiO_2_ anatase (*P*2_1_/*c*, 14), TiO_2_ rutile (*P*4_2_/*mnm*, 136), SnO_2_ (*P*4_2_/*mnm*, 136), CeO_2_ (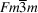, 225), Al_2_O_3_ (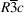, 167), LiMnO_2_ (*Pmmn*, 59), LiNiO_2_ (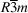, 166) and NaFeO_2_ (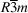, 166), where the space group in parentheses is the one that gives the best-fit result of the Rietveld analysis. The six SpgVolNet models did not unanimously assign the correct space group to Na_2/3_(Mn_2/3_Ni_1/3_)O_2_. The ensemble averaging technique nonetheless led to successful assignment of its correct space group as *P*6_3_/*mmc* (194), on which we elaborate in Section 4.2.2. These results demonstrate that the SpgVolNet models trained in the ideal world fared quite well in the real world. On the other hand, the SpgVolNet models assigned a space group of higher symmetry than the correct one to the remaining two materials, Fe_2_O_3_ (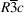, 167) and Li_2_MnO_3_ (*C*2/*m*, 12). For the former, the XRD profile has a very high background that blurs the micro-signals, which may be contributing to the error in the SpgVolNet model. The latter, Li_2_MnO_3_, epitomizes the intricate relationship between distinct space groups that are closely connected from a crystallographic viewpoint, as discussed in Section 4.2.3.

#### 4.2.2. Na_2/3_(Mn_2/3_Ni_1/3_)O_2_

**Figure 4:**
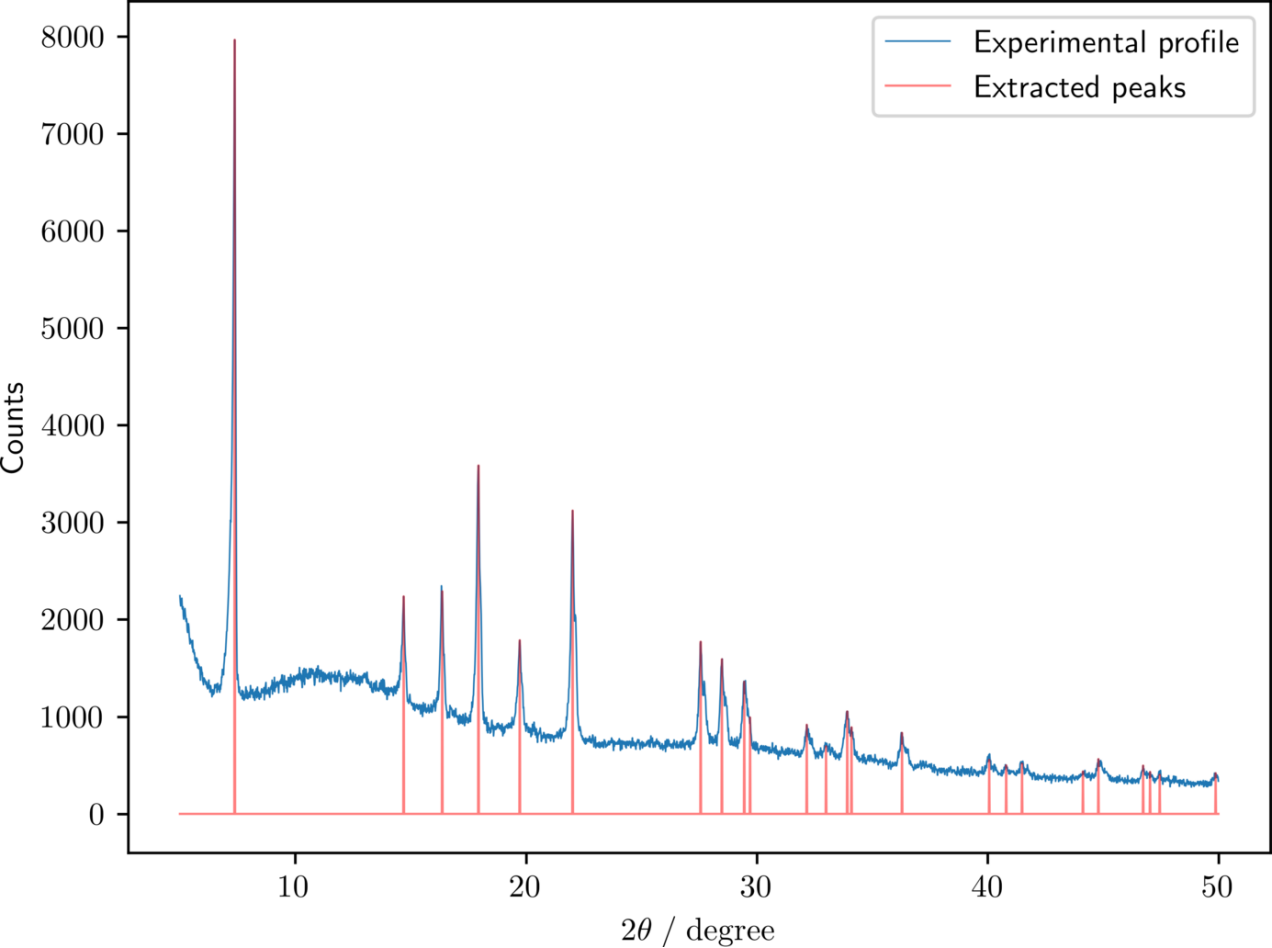
Experimental diffraction profile of Na_2/3_(Mn_2/3_Ni_1/3_)O_2_ and the extracted peaks. The diffraction profile was measured with an X-ray diffractometer (X’pert Pro, Malvern Panalytical) using Mo *K*α radiation (60 kV, 50 mA). The diffraction profile was recorded at a scanning rate of 1.5° min^−1^ in the 2θ range of 5–50°.

Fig. 4 ▸ shows the experimental XRD profile of the hexagonal compound Na_2/3_(Mn_2/3_Ni_1/3_)O_2_ and the extracted peak positions and intensities. Table 4 ▸ lists the space groups *g* to which the top three probabilities were assigned by the six SpgVolNet models, *p*_*m*_(*g*) (*m* = I–VI), and the ensemble mean probabilities, 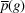 defined in equation (15), for this material. Unlike for the other ten materials tested in this study, the votes of each SpgVolNet model were significantly split for this material; the probability *p*_*m*_ of only one model (V) exceeded 90%. This material is reported to belong to *P*6_3_/*mmc*, a hexagonal crystal system, and our Rietveld analysis was also successful for this space group (see Fig. S14). However, as the votes were split among other space groups, we carried out a Le Bail refinement, which requires as input parameters only the space group and unit-cell constants (for the latter, the unit-cell volume predicted by SpgVolNet is helpful). The refinement under *P*6_3_/*mmc* successfully matched all the experimental diffraction peaks (see Fig. S15) and the reliability factors converged to levels comparable to those of the Rietveld analysis. On the other hand, the reliability factor *R*_wp_ never fell below 17 under 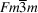 and 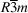, to which several SpgVolNet models assigned a certain level of probability. Model V confidently assigned the correct space group, *P*6_3_/*mmc* (194), with *p*_V_(*P*6_3_/*mmc*) = 0.94, followed by model IV with *p*_IV_(*P*6_3_/*mmc*) = 0.54. Models II and VI gave the second highest probabilities to *P*6_3_/*mmc*: *p*_II_(*P*6_3_/*mmc*) = 0.27 and *p*_VI_(*P*6_3_/*mmc*) = 0.27. What differentiates models I–VI is merely the randomizing seed to shuffle the common training data set. The overall prediction accuracies of all six models thus trained are comparable and reproducible (see Table SXI in the supporting information). Even if we obtain a CNN model with acceptable statistical metrics, the randomizing process of the data set could infuse a certain individuality into the model. The ensemble averaging technique managed to rank the correct answer, *P*6_3_/*mmc*, at the top of the prediction list, albeit with a relatively low probability, 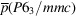 = 0.36, indicating the efficacy of this technique. This result also points to the possibility of using the space group and unit-cell volume predicted by the SpgVolNet models as a starting point for Le Bail refinement.

Unlike the other ten experimental XRD profiles which were measured using Cu *K*α radiation, the profile in Fig. 4 ▸ was measured using Mo *K*α radiation. Our strategy mentioned in the *Introduction*[Sec sec1] was rewarded, *i.e.* because the present CNN models were trained in the ideal world where the input data are independent of the experimental setup, we can obviate the need for re-training the models even if the experimental conditions vary.

#### 4.2.3. Li_2_MnO_3_

**Figure 5:**
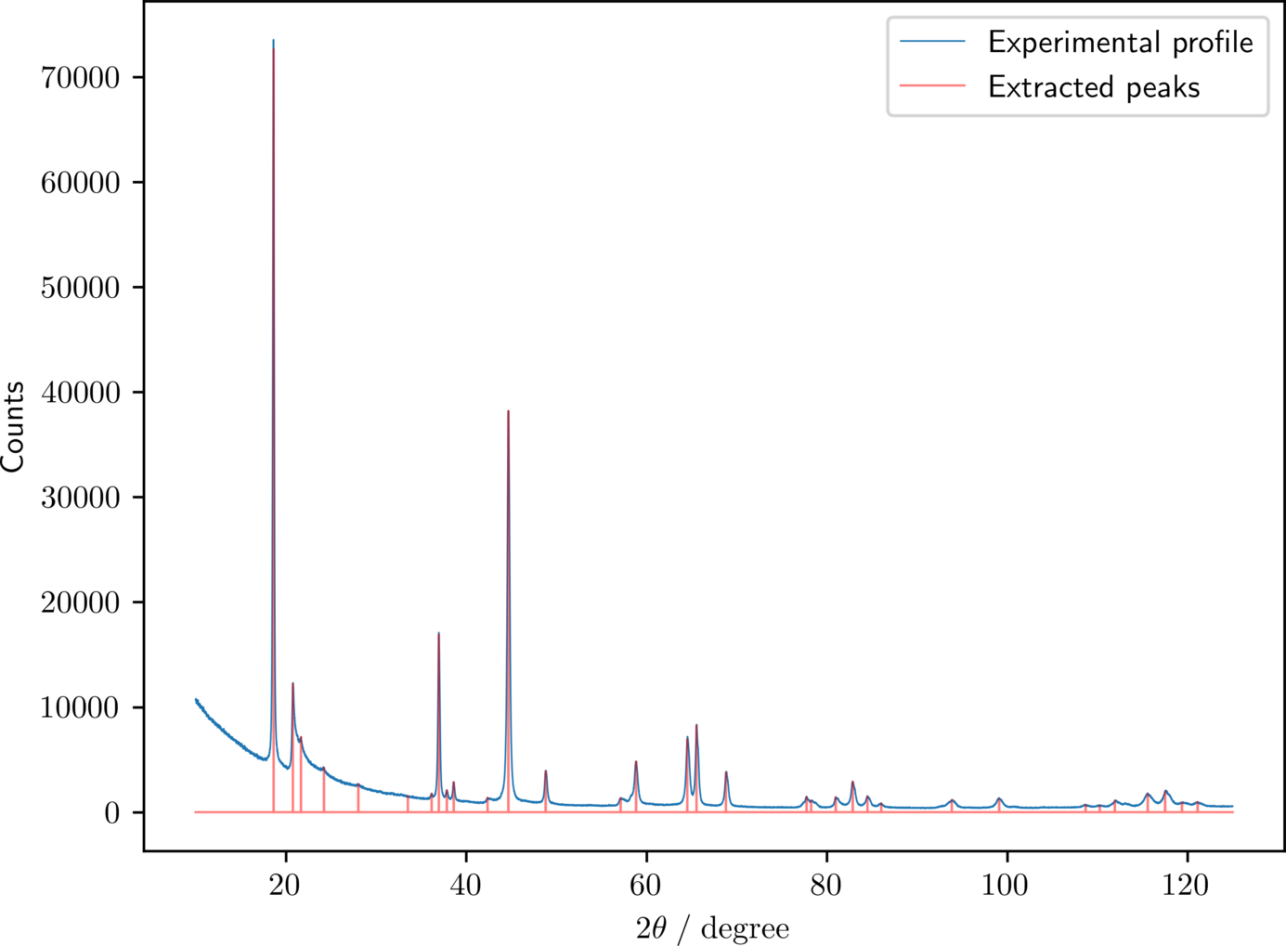
Experimental diffraction profile of Li_2_MnO_3_ and the extracted peaks. The diffraction profile was measured with an X-ray diffractometer (RINT-TTRIII, Rigaku) using Cu *K*α radiation (15 kV, 300 mA). The diffraction profile was recorded at a scanning rate of 2.0° min^−1^ in the 2θ range of 10–125°.

Table 5 ▸ summarizes the results of the space group classification for Li_2_MnO_3_ based on the peaks extracted from the experimental diffraction profile shown in Fig. 5 ▸. The compound expressed by the formula Li_2_MnO_3_ is generally considered to belong to *C*2/*m* (Strobel & Lambert-Andron, 1988 ▸; Thackeray *et al.*, 2007 ▸). For the present specific material, the SpgVolNet models unanimously suggested 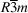.

What lies behind this discrepancy? Crystallographic scrutiny is required to answer this question. One has first to notice a group–subgroup relationship amongst the following space groups in terms of their symmetric operations: 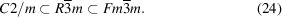
If all Li and Mn atoms in Li_2_MnO_3_ are completely disordered (*i.e.* indistinguishable), the structure has the highest symmetry, 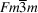, the rock salt structure or NaCl-type. From this rock salt structure, if three-quarters of the Li atoms and the remainder (one-quarter of Li + all Mn atoms) form alternating layers, the symmetry is broken into 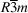, into which the SpgVolNet models confidently classified the present material.

This structure is often referred to as the ordered (or layered) rock salt structure, of which a representative material is LiNiO_2_, one of the eleven inorganic materials considered in this study (see Section S1.8 in the supporting information). In this case, the formula can be written as Li(Li_1/3_Mn_2/3_)O_2_, where the Li and Mn atoms in the layer of (Li_1/3_Mn_2/3_) are still completely disordered. When the Li and Mn atoms in this (Li_1/3_Mn_2/3_) layer are periodically ordered in a 1:2 ratio, the symmetry further drops to *C*2/*m*. Therefore, a substance expressed by the formula Li_2_MnO_3_ can belong to either of these distinct space groups, depending on the extent of the order–disorder in the Li and Mn arrangement. In fact, Rietveld analysis of the present experimental diffraction profile was equally well achieved by constraining the structure to be either 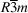 (Fig. S11) or *C*2/*m* (Fig. S10).

When the SpgVolNet models were tested with the peak intensities and their positions extracted from these two Rietveld-simulated profiles, instead of the peaks extracted from the experimental profile, they exclusively assigned the exact space group, namely 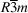 for the former and *C*2/*m* for the latter (see Table SX), suggesting that the prediction would have been correct if the diffraction profile were perfect. In that sense, training the SpgVolNet models in the ideal world (Scheme 1 mentioned in the *Introduction*[Sec sec1]) was successful. Although the cause of the discrepancy is not identifiable at present, three possibilities are conceivable: (i) the present material is in the most ordered state (*C*2/*m*) but the SpgVolNet models were misled by the low signal-to-noise ratio of the experimental data, which resulted in overlooking the small signals at high 1/*d*, the problem pertinent to Scheme 2 mentioned in the *Introduction*[Sec sec1]; and/or (ii) the SpgVolNet models erred on the side of a supergroup because considerable stacking faults in the material broadened some related diffraction peaks and lowered their intensities (Boulineau *et al.*, 2010 ▸); and/or (iii) the present material partially remains in a less ordered state in the (Li_1/3_Mn_2/3_) layer, *i.e.* in a higher-symmetry group, presumably because of kinetic effects during the material preparation.

Another example that potentially leads SpgVolNet to assign supergroups would be perovskite, CaTiO_3_. The perovskite-type compounds represented by CaTiO_3_ undergo a series of structural phase transitions, passing through the closely related space groups as (Ali & Yashima, 2005 ▸)
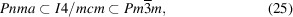
yet their diffraction profiles strongly resemble each other. Hence, if the SpgVolNet models are tested with an experimental diffraction profile of this compound, they might assign a supergroup of the correct space group, depending on the state of the material and/or the quality of the experimental data. From the viewpoint of experimentalists, the fact that the SpgVolNet models assigned a supergroup (or a higher symmetry group) is rather suggestive in understanding the state of the specific material that they synthesized, possibly in contrast to the general material found in the literature.

As a future task, we may be able to derive the correct space group from peak intensity anomalies, even in the presence of stacking faults or partial disorder, by taking into account the group–subgroup relationship. Such an approach is beyond the reach of conventional schemes, *e.g.* the Hanawalt method, for which anomalies in peak intensities are fatal.

#### 4.2.4. Cell volume prediction

The overall mean results of the six SpgVolNet models, I–VI, trained by five training data sets, 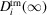 (*i* = 1–5), are presented (6 × 5 = 30 prediction results in total). As discussed in Section 4.2.3, the compound Li_2_MnO_3_ prepared in this study likely belongs to either *C*2/*m* or 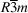; hence, the correct cell volume can be either *V*(*C*2/*m*) or 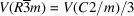.

Table 6 ▸ summarizes the results of cell volume prediction. Among the eleven materials tested, six of them (TiO_2_ anatase, Fe_2_O_3_, CeO_2_, Al_2_O_3_, LiMnO_2_ and LiNiO_2_) were predicted in the range indicated in equation (23), consistent with the fact that 50% of the structures in the validation data sets are in this range.

Although higher-symmetry space groups were assigned to Fe_2_O_3_, its cell volume was correctly predicted. Conversely, while the space group was correctly assigned to TiO_2_ rutile (*P*4_2_/*mnm*), its unit-cell volume was overestimated by more than 40%. At present, we cannot decisively pinpoint the cause, yet impurities in the present material may have affected the volume prediction. As discussed in Section 4.2.3, the SpgVolNet models judged the Li_2_MnO_3_ sample to have space group 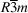. The SpgVolNet models overestimated the ‘correct’ cell volume by only 9% on the basis of this judgement.

## 5. Conclusions

In extracting the structural information of inorganic materials from their diffraction profiles using the deep learning approach, this study aimed to achieve the following two objectives: (i) to construct CNN models in the ideal world and to test them in the real world, and (ii) to examine the effect of the ensemble averaging technique and the multi-task learning approach. We summarize the findings below in reverse order.

### 5.1. Ensemble averaging and multi-task learning

From each of the five training data sets, 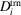 (1 ≤ *i* ≤ 5), we created six CNN models, I–VI, by shuffling the data set. The analysis of the confusion matrix in Section 4.1.1 revealed that the ensemble averaging technique improves the statistical metrics, especially the precision, as suggested by 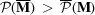, *i.e.* the technique suppresses false positive cases. In contrast, the MTL approach, in which the space group classification is coupled with cell volume prediction, improves the recall 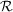 by reducing false negative cases. These two methods can hence complimentarily improve the generalization performance of multi-class classification problems. The effectiveness of ensemble averaging was demonstrated when the SpgVolNet models were tested using the experimental XRD profile of Na_2/3_(Mn_2/3_Ni_1/3_)O_2_ in Section 4.2.2. While not all six SpgVolNet models correctly classified this hexagonal compound, the ensemble-averaged outputs led to the correct answer, *P*6_3_/*mmc*. The SpgVolNet models predicted the cell volumes of eleven real samples from their experimental XRD profiles with a precision accuracy consistent with the validation results in equation (23).

### 5.2. Train the models in the ideal world and test them in the real world

In assigning the space group and estimating the primitive cell volume of an inorganic crystal from its XRD profile through the deep learning approach, we split the process into two schemes (see the *Introduction*[Sec sec1]): Scheme 1 trains the CNN models with a theoretical δ-function profile that reflects the intrinsic properties of a crystal structure of infinite crystal size, and Scheme 2 extracts relevant signals from the experimental XRD profile to feed the CNN models after conversion. The present study demonstrated that the SpgVolNet models trained by the ideal profiles derived only from the intrinsic properties of the crystal structure can successfully forecast the space group and predict the cell volume from the real experimental profiles. Hence the extrinsic experimental conditions, such as the wavelength of the incident X-ray beam, line broadening caused by the finite size or other specific conditions of the specimen, including the signal-to-noise ratio of the apparatus, have a limited effect.

The advantage of our strategy of separating Schemes 1 and 2 was demonstrated by testing the SpgVolNet models with experimental profiles measured using not only Cu *K*α radiation but also Mo *K*α radiation (Section 4.2.2.) As discussed in Section 4.2.3, where the SpgVolNet models were tested with the experimental data of Li_2_MnO_3_, the models did not classify the space group into *C*2/*m*, to which this compound is generally considered to belong. Instead, they assigned its minimal supergroup 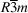, leaving the possibility that the specific specimen synthesized in this study is not in a perfectly ordered state, *C*2/*m*. However, the SpgVolNet models assigned *C*2/*m* to the hypothetical profile that was fitted to the experimental XRD profile of Li_2_MnO_3_ by constraining the symmetry to be *C*2/*m*. This observation exemplifies the situation where the mismatch does not stem from Scheme 1 (the CNN models trained in the ideal world) but rather from Scheme 2 (the difficulty of extracting adequate signals) and/or from the properties of the specific material (it is in a less ordered phase).

The CNN models trained on diffraction profiles with an infinite signal-to-noise ratio, free from noise and the effects of line shape, guide us in the direction of further improving the deep learning technique. For example, by systematically observing how the signal-to-noise ratio and line shape influence the CNN models trained without considering these factors, we may be able to let the models correct their tentative answers. Improving Scheme 2 is also a crucial step in accurately reconstructing the structural information from the diffraction profile, *e.g.* reducing noise, detecting impurities or identifying multiple phases, wherein another deep learning approach will play a significant role.

## Supplementary Material

Additional figures and tables. DOI: 10.1107/S1600576725002419/vb5091sup1.pdf

Data repository for the pre-trained models, data sets and test data: https://doi.org/10.57765/2003342

## Figures and Tables

**Table 1:** The 57 frequent space groups in the PowCod database and their number of entries

Space group (No.)	No. of entries
*P*2_1_/*c* (14)	7982
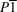 (2)	5897
*Pnma* (62)	4707
*C*2/*c* (15)	4203
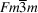 (225)	3955
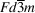 (227)	3463
*C*2/*m* (12)	2541
*P*6_3_/*mmc* (194)	2010
*I*4/*mmm* (139)	1807
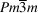 (221)	1714
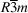 (166)	1287
*Cmcm* (63)	1248
*P*6/*mmm* (191)	999
*P*2_1_/*m* (11)	992
*Pbca* (61)	924
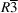 (148)	921
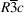 (167)	880
*P*6_3_/*m* (176)	876
*P*4/*mmm* (123)	794
*P*2_1_2_1_2_1_ (19)	693
*P*4/*nmm* (129)	654
*Pna*2_1_ (33)	632
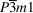 (164)	626
*P*2_1_ (4)	615
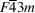 (216)	591
*P*1 (1)	574
*Cc* (9)	507
*I*4_1_/*amd* (141)	491
*I*4/*mcm* (140)	479
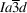 (230)	469
*P*4_2_/*mnm* (136)	467
*Pbcn* (60)	464
*P*2/*c* (13)	429
*Pbam* (55)	428
*P*6_3_*mc* (186)	398
*I*4_1_/*a* (88)	395
*Pnnm* (58)	395
*Cmc*2_1_ (36)	394
*P*6_3_ (173)	388
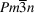 (223)	383
*C*2 (5)	381
*Immm* (71)	378
*Cmca* (64)	377
*I*4/*m* (87)	363
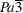 (205)	355
*Pmmm* (47)	328
*Imma* (74)	322
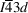 (220)	317
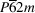 (189)	316
*P*2_1_3 (198)	287
*Pmmn* (59)	270
*R*3*m* (160)	260
*P*4/*mbm* (127)	255
*P*6_3_/*mcm* (193)	238
*Cmmm* (65)	237
*R*3 (146)	237
*R*3*c* (161)	237
Total	62830

**Table 2:** The number of entries in the data sets after excluding outliers from the parent data set *D*(∞), which consists of crystallographic structures belonging to any of the 57 space groups listed in Table 1

Data set	No. of entries
*D*(∞)	62830
*D*(2σ_η_)	60550
*D*(2σ_*U*_)	59701
*D*(2σ_*U*+η_)	57576
*D*(σ_η_)	44248
*D*(σ_*U*_)	43896
*D*(σ_*U*+η_)	31041

**Table 3:** Precision 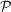, recall 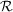 and *F*-score 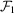 of SpgNet (single-task learning) and SpgVolNet (multi-task learning)

Metric	SpgNet (%)	SpgVolNet (%)
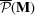	80.18 ± 0.41	80.42 ± 0.42
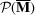	82.22 ± 0.45	82.22 ± 0.87
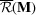	70.99 ± 0.52	72.14 ± 0.52
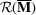	72.1 ± 1.2	73.6 ± 1.3
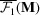	72.30 ± 0.55	73.23 ± 0.57
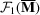	73.7 ± 1.3	74.9 ± 1.4

**Table 4:** Space group (and its probability in parentheses) predicted by the six SpgVolNet models (I–VI) and their ensemble mean (EM) based on the peaks extracted from the experimental diffraction profile of Na_2/3_(Mn_2/3_Ni_1/3_)O_2_ shown in Fig. 4 Rietveld refinement gives the best fit with *P*6_3_/*mmc* (194).

	I	II	III	IV	V	VI	EM
1	225 (30%)	166 (52%)	166 (27%)	194 (54%)	194 (94%)	141 (60%)	194 (36%)
2	166 (20%)	194 (27%)	225 (23%)	225 (21%)	225 (5%)	194 (27%)	166 (18%)
3	221 (15%)	225 (13%)	160 (22%)	191 (9%)	221 (1%)	186 (12%)	225 (15%)

**Table 5:** Space group (and its probability in parentheses) predicted by the six SpgVolNet models (I–VI) and their ensemble mean (EM) based on the peaks extracted from the experimental diffraction profile of Li_2_MnO_3_ shown in Fig. 5 A probability of 0% means it is below 0.5%. Rietveld analysis suggests that this specimen belongs to *C*2/*m* (12), of which a minimal supergroup is 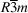 (166).

	I	II	III	IV	V	VI	EM
1	166 (100%)	166 (100%)	166 (100%)	166 (100%)	166 (100%)	166 (100%)	166 (100%)
2	160 (0%)	160 (0%)	160 (0%)	148 (0%)	160 (0%)	160 (0%)	160 (0%)
3	148 (0%)	148 (0%)	148 (0%)	160 (0%)	227 (0%)	146 (0%)	148 (0%)

**Table 6:** Predicted (*V*′) and correct (*V*) primitive cell volume

	*V*′ (Å^3^)	*V* (Å^3^)	(*V*′/*V*) − 1 (%)
TiO_2_ anatase	69.2	68.2	1.5
TiO_2_ rutile	88.9	62.5	42.3
Fe_2_O_3_	98.3	100.7	−2.4
SnO_2_	69.2	71.8	−3.7
CeO_2_	39.8	39.6	0.4
Al_2_O_3_	83.2	85.1	−2.2
Li_2_MnO_3_ (*C*2/*m*)†	36.3	100.0	−63.7
Li_2_MnO_3_ ( 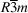 )†	36.3	33.3	9.0
LiMnO_2_	72.8	73.7	−1.3
LiNiO_2_	33.8	33.9	−0.4
NaFeO_2_	46.8	42.6	9.8
Na_2/3_(Mn_2/3_Ni_1/3_)O_2_	69.0	79.1	−12.7

† For these materials, the correct answer could be either *V*(*C*2/*m*) or 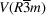 = *V*(*C*2/*m*)/3 (see text).

## Data Availability

SpgVolNet, with the pre-trained models, data sets and XRD profiles extracted from experimental data, is available at https://doi.org/10.57765/2003342.

## References

[bb1] Ali, R. & Yashima, M. (2005). *J. Solid State Chem.* **178**, 2867–2872.

[bb2] Altomare, A., Corriero, N., Cuocci, C., Falcicchio, A., Moliterni, A. & Rizzi, R. (2015). *J. Appl. Cryst.* **48**, 598–603.

[bb3] Boulineau, A., Croguennec, L., Delmas, C. & Weill, F. (2010). *Solid State Ionics*, **180**, 1652–1659.

[bb4] Breiman, L. (1996). *Mach. Learn.* **24**, 123–140.

[bb5] Corriero, N., Rizzi, R., Settembre, G., Del Buono, N. & Diacono, D. (2023). *J. Appl. Cryst.* **56**, 409–419. 10.1107/S1600576723000596 PMC10077848 37032966

[bb6] Du, P., Kibbe, W. A. & Lin, S. M. (2006). *Bioinformatics*, **22**, 2059–2065. 10.1093/bioinformatics/btl355 16820428

[bb7] Floridi, L. & Chiriatti, M. (2020). *Minds Machines*, **30**, 681–694.

[bb8] Gražulis, S., Chateigner, D., Downs, R. T., Yokochi, A. F. T., Quirós, M., Lutterotti, L., Manakova, E., Butkus, J., Moeck, P. & Le Bail, A. (2009). *J. Appl. Cryst.* **42**, 726–729. 10.1107/S0021889809016690 PMC3253730 22477773

[bb9] Hansen, L. & Salamon, P. (1990). *IEEE Trans. Pattern Anal. Mach. Intell.* **12**, 993–1001.

[bb10] Hendrycks, D. & Gimpel, K. (2017). *International conference on**learning representations*, https://openreview.net/forum?id=Hkg4TI9xl.

[bb13] Hjorth Larsen, A., Jørgen Mortensen, J., Blomqvist, J., Castelli, I. E., Christensen, R., Dułak, M., Friis, J., Groves, M. N., Hammer, B., Hargus, C., Hermes, E. D., Jennings, P. C., Bjerre Jensen, P., Kermode, J., Kitchin, J. R., Leonhard Kolsbjerg, E., Kubal, J., Kaasbjerg, K., Lysgaard, S., Bergmann Maronsson, J., Maxson, T., Olsen, T., Pastewka, L., Peterson, A., Rostgaard, C., Schiøtz, J., Schütt, O., Strange, M., Thygesen, K. S., Vegge, T., Vilhelmsen, L., Walter, M., Zeng, Z. & Jacobsen, K. W. (2017). *J. Phys. Condens. Matter*, **29**, 273002. 10.1088/1361-648X/aa680e 28323250

[bb11] Ibers, J. A. & Hamilton, W. C. (1974). Editors. *International tables for X-ray crystallography*, Vol. IV. Birmingham: Kynoch Press.

[bb12] Lakshminarayanan, B., Pritzel, A. & Blundell, C. (2017). *Advances in neural information processing systems*, edited by I. Guyon, U. V. Luxburg, S. Bengio, H. Wallach, R. Fergus, S. Vishwanathan & R. Garnett, Vol. 30. https://proceedings.neurips.cc/paper_files/paper/2017/file/9ef2ed4b7fd2c810847ffa5fa85bce38-Paper.pdf. Red Hook: Curran Associates.

[bb14] Liang, H., Stanev, V., Kusne, A. G. & Takeuchi, I. (2020). *Phys. Rev. Mater.* **4**, 123802.

[bb15] Liu, C.-H., Tao, Y., Hsu, D., Du, Q. & Billinge, S. J. L. (2019). *Acta Cryst.* A**75**, 633–643. 10.1107/S2053273319005606 31264647

[bb16] Lolla, S., Liang, H., Kusne, A. G., Takeuchi, I. & Ratcliff, W. (2022). *J. Appl. Cryst.* **55**, 882–889. 10.1107/S1600576722006069 PMC9348870 35974721

[bb18] Nair, V. & Hinton, G. E. (2010). *The 27th international conference on machine learning (ICML)*, pp. 807–814. https://icml.cc/Conferences/2010/papers/432.pdf. Omnipress.

[bb19] Ong, S. P., Richards, W. D., Jain, A., Hautier, G., Kocher, M., Cholia, S., Gunter, D., Chevrier, V. L., Persson, K. A. & Ceder, G. (2013). *Comput. Mater. Sci.* **68**, 314–319.

[bb20] Ovadia, Y., Fertig, E., Ren, J., Nado, Z., Sculley, D., Nowozin, S., Dillon, J. V., Lakshminarayanan, B. & Snoek, J. (2019). *Advances in neural information processing systems*, edited by H. Wallach, H. Larochelle, A. Beygelzimer, F. d’Alché-Buc, E. Fox & R. Garnett, Vol. 32. https://proceedings.neurips.cc/paper_files/paper/2019/file/8558cb408c1d76621371888657d2eb1d-Paper.pdf. Red Hook: Cur­ran Associates.

[bb21] Oviedo, F., Ren, Z., Sun, S., Settens, C., Liu, Z., Hartono, N. T. P., Ramasamy, S., DeCost, B. L., Tian, S. I. P., Romano, G., Gilad Kusne, A. & Buonassisi, T. (2019). *npj Comput. Mater.* **5**, 60.

[bb22] Park, W. B., Chung, J., Jung, J., Sohn, K., Singh, S. P., Pyo, M., Shin, N. & Sohn, K.-S. (2017). *IUCrJ*, **4**, 486–494. 10.1107/S205225251700714X PMC5571811 28875035

[bb23] Paszke, A., Gross, S., Chintala, S., Chanan, G., Yang, E., DeVito, Z., Lin, Z., Desmaison, A., Antiga, L. & Lerer, A. (2017). *31st conference on neural information processing systems (NIPS)*, https://openreview.net/forum?id=BJJsrmfCZ.

[bb24] Rombach, R., Blattmann, A., Lorenz, D., Esser, P. & Ommer, B. (2022). *2022 IEEE/CVF conference on computer vision and pattern recognition (CVPR)*, pp. 10674–10685. IEEE.

[bb25] Sener, O. & Koltun, V. (2018). *32st conference on neural information processing systems (NeurIPS)*, https://proceedings.neurips.cc/paper_files/paper/2018/file/432aca3a1e345e339f35a30c8f65edce-Paper.pdf.

[bb26] Setyawan, W. & Curtarolo, S. (2010). *Comput. Mater. Sci.* **49**, 299–312.

[bb27] Silver, D., Huang, A., Maddison, C. J., Guez, A., Sifre, L., van den Driessche, G., Schrittwieser, J., Antonoglou, I., Panneershelvam, V., Lanctot, M., Dieleman, S., Grewe, D., Nham, J., Kalchbrenner, N., Sutskever, I., Lillicrap, T., Leach, M., Kavukcuoglu, K., Graepel, T. & Hassabis, D. (2016). *Nature*, **529**, 484–489. 10.1038/nature16961 26819042

[bb28] Stehman, S. V. (1997). *Remote Sens. Environ.* **62**, 77–89.

[bb29] Strobel, P. & Lambert-Andron, B. (1988). *J. Solid State Chem.* **75**, 90–98.

[bb30] Thackeray, M. M., Kang, S.-H., Johnson, C. S., Vaughey, J. T., Benedek, R. & Hackney, S. A. (2007). *J. Mater. Chem.* **17**, 3112–3125.

[bb31] Togo, A., Shinohara, K. & Tanaka, I. (2018). *arXiv*, 1808.01590.

[bb32] Vecsei, P. M., Choo, K., Chang, J. & Neupert, T. (2019). *Phys. Rev. B*, **99**, 245120.

[bb33] Venkatraman, V. & Carvalho, P. A. (2022). *Acta Mater.* **240**, 118353.

[bb34] Xie, T. & Grossman, J. C. (2018). *Phys. Rev. Lett.* **120**, 145301. 10.1103/PhysRevLett.120.145301 29694125

[bb35] Ziletti, A., Kumar, D., Scheffler, M. & Ghiringhelli, L. M. (2018). *Nat. Commun.* **9**, 2775. 10.1038/s41467-018-05169-6 PMC6050314 30018362

[bb36] Zoph, B. & Le, Q. (2017). *International conference on learning representations*, https://openreview.net/forum?id=r1Ue8Hcxg.

