# Supplementary material for: Prediction of the space group and cell volume by training a convolutional neural network with primitive ‘ideal’ diffraction profiles and its application to ‘real’ experimental data
Source: J Appl Crystallogr. 2025 Apr 25;58(Pt 3):718–30. doi: 10.1107/S1600576725002419 (PMC12135985; doi:10.1107/S1600576725002419)
Supplement: Supplementary file 1 [file j-58-00718-sup1.pdf]

JOURNAL OF APPLIED CRYSTALLOGRAPHY

**Volume 58 (2025)**

**Supporting information for article:**

# Prediction of the space group and cell volume by training a convolutional neural network with primitive `ideal' diffraction profiles and its application to `real' experimental data

**Hiroyuki Ozaki, Naoya Ishida and Tetsu Kiyobayashi**

## S1. Space group classifications of the experimental diffraction profiles

### *S1.1. $TiO_2$ anatase*

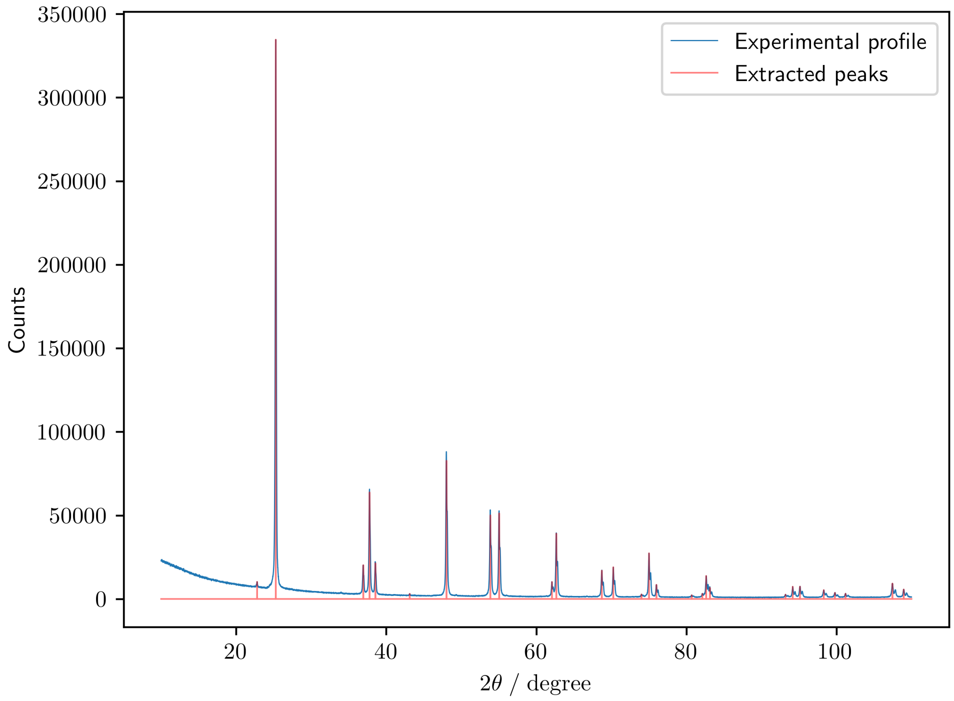


Fig. S1. Experimental diffraction profile of $TiO_2$ anatase and the extracted peaks. The diffraction profile was measured with an X-ray diffractometer (UltimaIV, Rigaku) using Cu-$K\alpha$ radiation (40 kV, 40 mA). The diffraction profile was recorded at a scanning rate of 4.0° / min in the $2\theta$ range of 10–110°.

Table SI. *Space group (and its probability in parentheses) predicted by the six SpgVolNet models (*I–VI*) and their ensemble mean (EM) based on the peaks extracted from the experimental diffraction profile of $TiO_2$ anatase shown in Fig. S1. The probability (0%) means it is below 0.5%. [Rietveld: $I4_1/amd$ (141)]*

| | I | II | III | IV | V | VI | EM |
|---|---|---|---|---|---|---|---|
| 1 | 141(100%) | 141(100%) | 141(100%) | 141(100%) | 141(100%) | 141(100%) | 141(100%) |
| 2 | 194(0%) | 173(0%) | 167(0%) | 194(0%) | 166(0%) | 166(0%) | 194(0%) |
| 3 | 173(0%) | 63(0%) | 186(0%) | 191(0%) | 194(0%) | 194(0%) | 191(0%) |

### S1.2. $TiO_2$ rutile

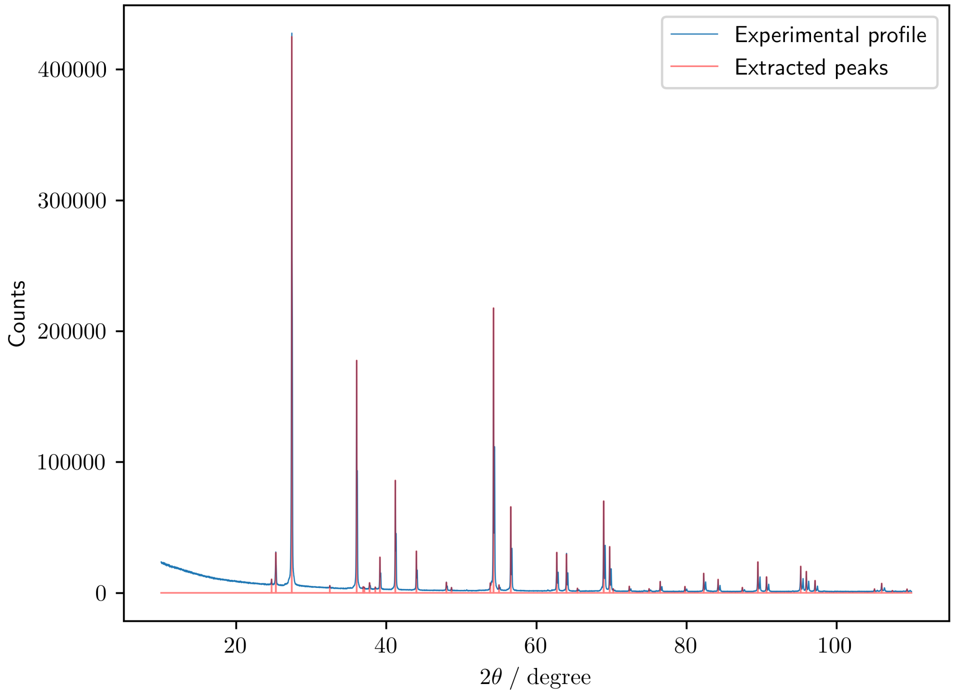


Fig. S2. Experimental diffraction profile of $TiO_2$ rutile and the extracted peaks. The diffraction profile was measured with an X-ray diffractometer (UltimaIV, Rigaku) using Cu-$K\alpha$ radiation (40 kV, 40 mA). The diffraction profile was recorded at a scanning rate of 4.0° / min in the $2\theta$ range of 10–110°.

Table SII. *Space group (and its probability in parentheses) predicted by the six SpgVolNet models (*I–VI*) and their ensemble mean (EM) based on the peaks extracted from the experimental diffraction profile of $TiO_2$ rutile shown in Fig. S2. The probability (0%) means it is below 0.5%. [Rietveld: $P4_2/mnm$ (136)]*

| | I | II | III | IV | V | VI | EM |
|---|---|---|---|---|---|---|---|
| 1 | 136(98%) | 136(66%) | 136(100%) | 136(100%) | 136(100%) | 136(100%) | 136(94%) |
| 2 | 141(1%) | 14(20%) | 141(0%) | 141(0%) | 167(0%) | 141(0%) | 14(3%) |
| 3 | 167(0%) | 58(5%) | 167(0%) | 58(0%) | 141(0%) | 139(0%) | 58(1%) |

*S1.3. $Fe_2O_3$*

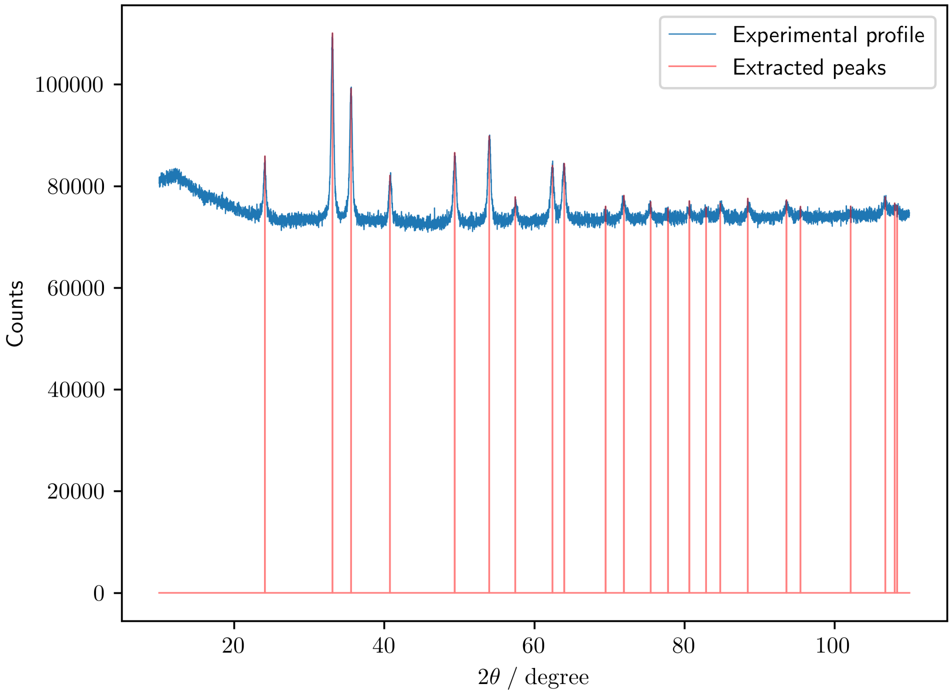


Fig. S3. Experimental diffraction profile of $Fe_2O_3$ and the extracted peaks. The diffraction profile was measured with an X-ray diffractometer (UltimaIV, Rigaku) using Cu-$K\alpha$ radiation (40 kV, 40 mA). The diffraction profile was recorded at a scanning rate of 4.0° / min in the $2\theta$ range of 10–110°.

Table SIII. *Space group (and its probability in parentheses) predicted by the six SpgVolNet models (I–VI) and their ensemble mean (EM) based on the peaks extracted from the experimental diffraction profile of $Fe_2O_3$ shown in Fig. S3. [Rietveld: $R\bar{3}c$ (167)]*

| | I | II | III | IV | V | VI | EM |
|---|---|---|---|---|---|---|---|
| 1 | 220(85%) | 225(31%) | 221(74%) | 223(79%) | 139(44%) | 220(27%) | 220(24%) |
| 2 | 205(7%) | 216(25%) | 225(14%) | 225(12%) | 220(18%) | 191(16%) | 223(18%) |
| 3 | 225(4%) | 220(15%) | 191(3%) | 227(4%) | 223(17%) | 221(15%) | 221(17%) |

### *S1.4. $SnO_2$*

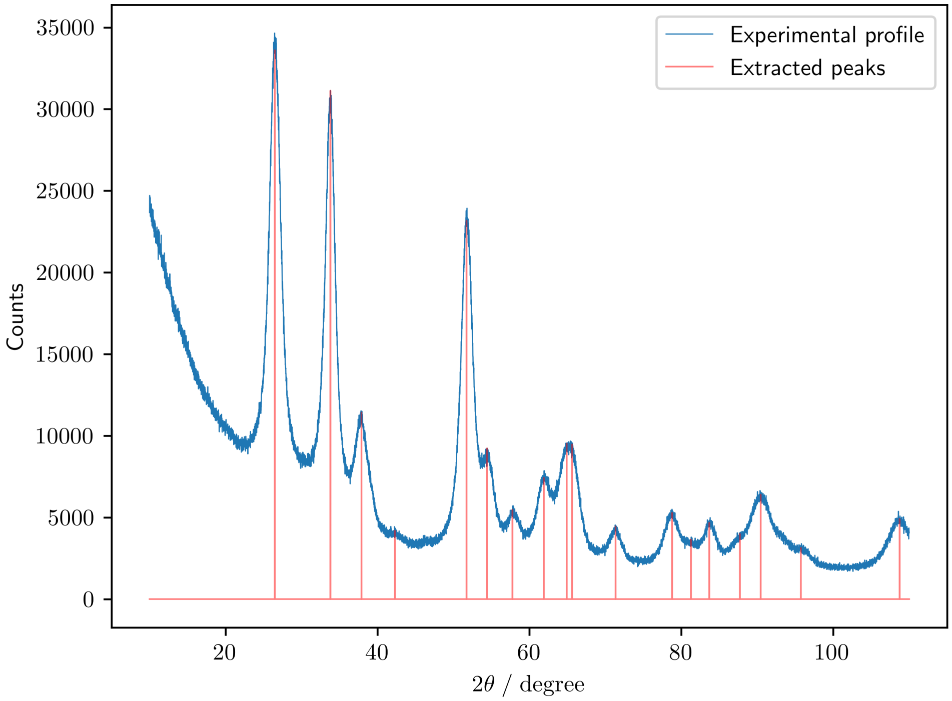


Fig. S4. Experimental diffraction profile of $SnO_2$ and the extracted peaks. The diffraction profile was measured with an X-ray diffractometer (UltimaIV, Rigaku) using Cu-$K\alpha$ radiation (40 kV, 40 mA). The diffraction profile was recorded at a scanning rate of 4.0° / min in the $2\theta$ range of 10–110°.

Table SIV. *Space group (and its probability in parentheses) predicted by the six SpgVolNet models (I–VI) and their ensemble mean (EM) based on the peaks extracted from the experimental diffraction profile of $SnO_2$ shown in Fig. S4. The probability (0%) means it is below 0.5%. [Rietveld: $P4_2/mnm$ (136)]*

| | I | II | III | IV | V | VI | EM |
|---|---|---|---|---|---|---|---|
| 1 | 136(100%) | 136(99%) | 136(100%) | 136(100%) | 136(100%) | 136(100%) | 136(100%) |
| 2 | 221(0%) | 141(0%) | 139(0%) | 221(0%) | 221(0%) | 139(0%) | 141(0%) |
| 3 | 205(0%) | 191(0%) | 191(0%) | 227(0%) | 58(0%) | 221(0%) | 191(0%) |

*S1.5. $CeO_2$*

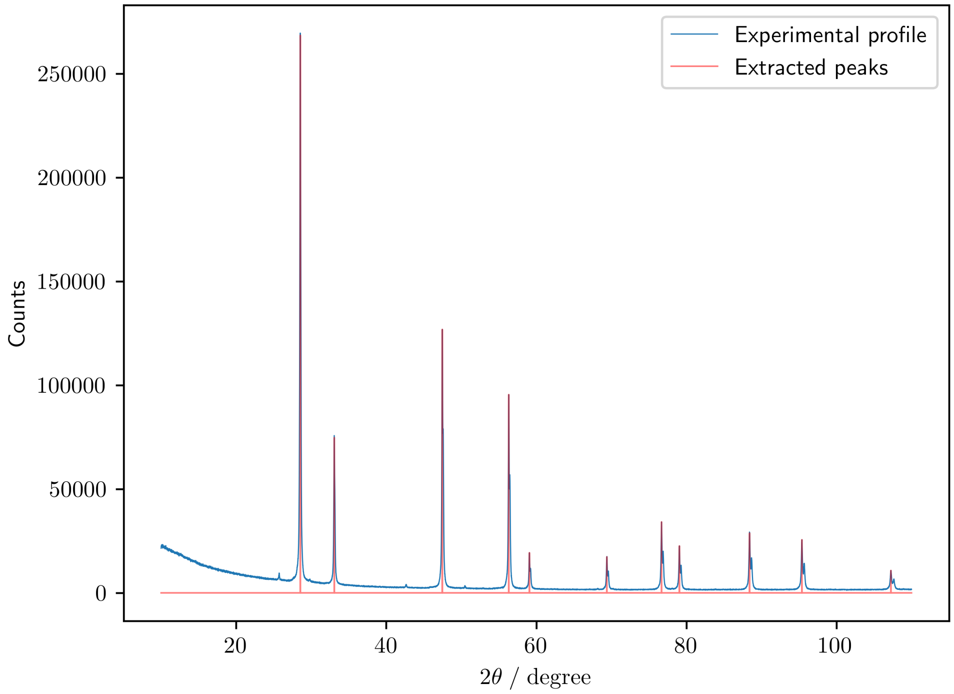


Fig. S5. Experimental diffraction profile of $CeO_2$ and the extracted peaks. The diffraction profile was measured with an X-ray diffractometer (UltimaIV, Rigaku) using Cu-$K\alpha$ radiation (40 kV, 40 mA). The diffraction profile was recorded at a scanning rate of 4.0° / min in the $2\theta$ range of 10–110°.

Table SV. *Space group (and its probability in parentheses) predicted by the six SpgVolNet models (I–VI) and their ensemble mean (EM) based on the peaks extracted from the experimental diffraction profile of $CeO_2$ shown in Fig. S5. The probability (0%) means it is below 0.5%. [Rietveld: $Fm\bar{3}m$ (225)]*

| | I | II | III | IV | V | VI | EM |
|---|---|---|---|---|---|---|---|
| 1 | 225(97%) | 225(97%) | 225(98%) | 225(93%) | 225(98%) | 225(96%) | 225(97%) |
| 2 | 216(3%) | 216(3%) | 216(2%) | 216(7%) | 216(2%) | 216(4%) | 216(3%) |
| 3 | 139(0%) | 139(0%) | 139(0%) | 139(0%) | 139(0%) | 139(0%) | 139(0%) |

*S1.6. $Al_2O_3$*

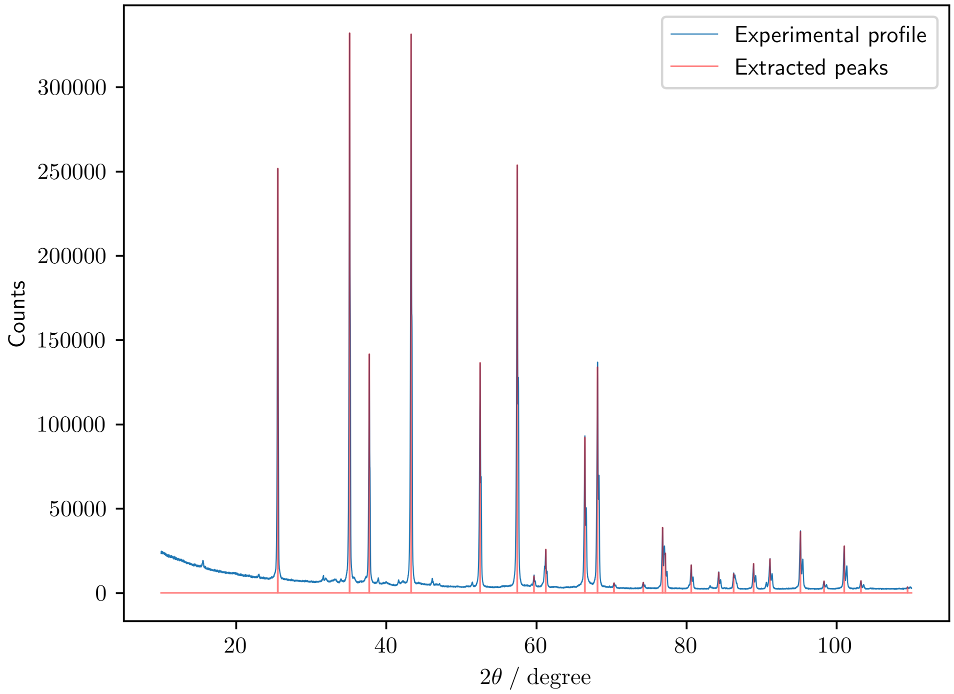


Fig. S6. Experimental diffraction profile of $Al_2O_3$ and the extracted peaks. The diffraction profile was measured with an X-ray diffractometer (UltimaIV, Rigaku) using Cu-$K\alpha$ radiation (40 kV, 40 mA). The diffraction profile was recorded at a scanning rate of 4.0° / min in the $2\theta$ range of 10–110°.

Table SVI. *Space group (and its probability in parentheses) predicted by the six SpgVolNet models (I–VI) and their ensemble mean (EM) based on the peaks extracted from the experimental diffraction profile of $Al_2O_3$ shown in Fig. S6. The probability (0%) means it is below 0.5%. [Rietveld: $R\bar{3}c$ (167)]*

| | I | II | III | IV | V | VI | EM |
|---|---|---|---|---|---|---|---|
| 1 | 167(100%) | 167(100%) | 167(100%) | 167(100%) | 167(100%) | 167(100%) | 167(100%) |
| 2 | 225(0%) | 223(0%) | 161(0%) | 161(0%) | 148(0%) | 161(0%) | 223(0%) |
| 3 | 148(0%) | 194(0%) | 148(0%) | 166(0%) | 161(0%) | 166(0%) | 161(0%) |

*S1.7. $LiMnO_2$*

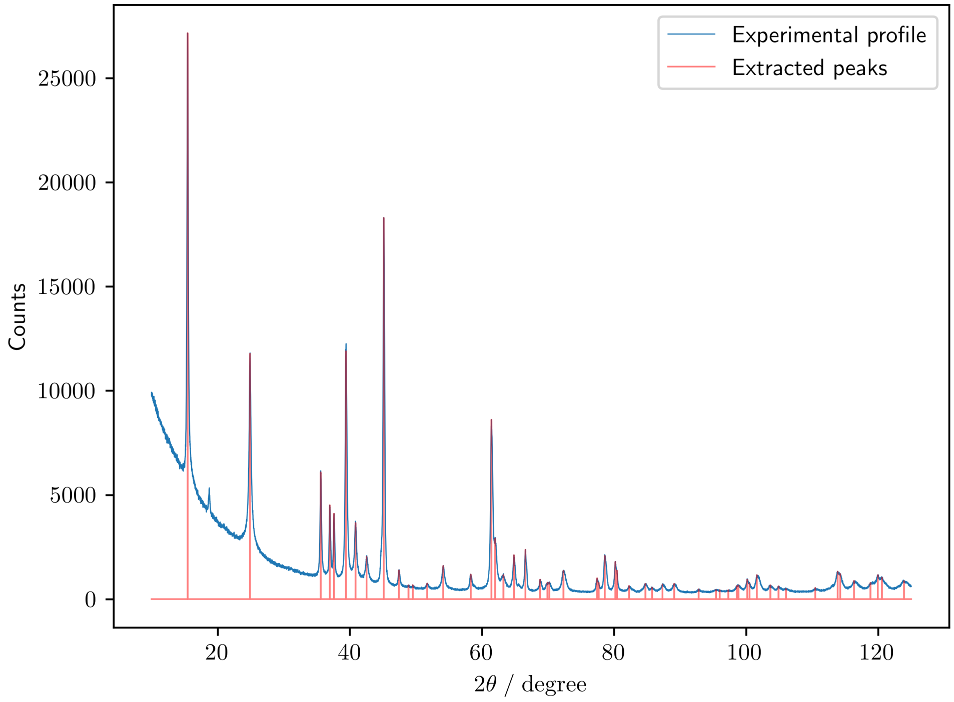


Fig. S7. Experimental diffraction profile of $LiMnO_2$ and the extracted peaks. The diffraction profile was measured with an X-ray diffractometer (RINT-TTRIII, Rigaku) using Cu-K$\alpha$ radiation (15 kV, 300 mA). The diffraction profile was recorded at a scanning rate of 2.0° / min in the $2\theta$ range of 10–125°.

Table SVII. *Space group (and its probability in parentheses) predicted by the six SpgVolNet models (*I–VI*) and their ensemble mean (EM) based on the peaks extracted from the experimental diffraction profile of $LiMnO_2$ shown in Fig. S7. The probability (0%) means it is below 0.5%. [Rietveld: Pmmn (59)]*

| | I | II | III | IV | V | VI | EM |
|---|---|---|---|---|---|---|---|
| 1 | 59(100%) | 59(100%) | 59(100%) | 59(100%) | 59(100%) | 59(100%) | 59(100%) |
| 2 | 140(0%) | 12(0%) | 62(0%) | 141(0%) | 194(0%) | 64(0%) | 194(0%) |
| 3 | 62(0%) | 193(0%) | 194(0%) | 12(0%) | 141(0%) | 13(0%) | 141(0%) |

### *S1.8. $LiNiO_2$*

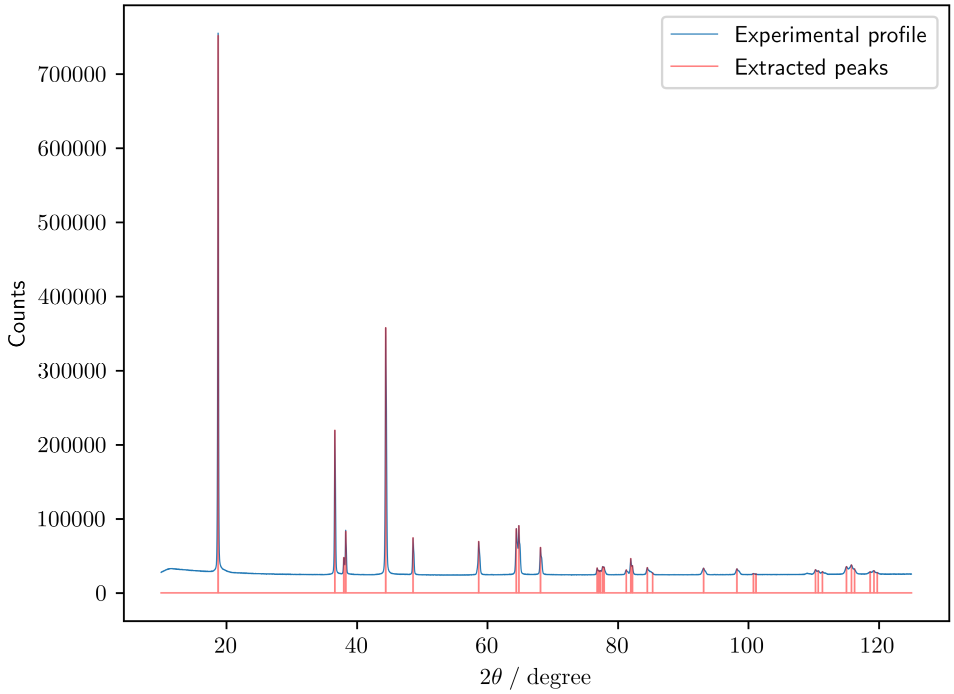


Fig. S8. Experimental diffraction profile of $LiNiO_2$ and the extracted peaks. The diffraction profile was measured with an X-ray diffractometer (RINT-TTRIII, Rigaku) using Cu-K$\alpha$ radiation (15 kV, 300 mA). The diffraction profile was recorded at a scanning rate of 2.0° / min in the $2\theta$ range of 10–125°.

Table SVIII. *Space group (and its probability in parentheses) predicted by the six SpgVolNet models (I–VI) and their ensemble mean (EM) based on the peaks extracted from the experimental diffraction profile of $LiNiO_2$ shown in Fig. S8. The probability (0%) means it is below 0.5%. [Rietveld: $R\bar{3}m$ (166)]*

| | I | II | III | IV | V | VI | EM |
|---|---|---|---|---|---|---|---|
| 1 | 166(100%) | 166(100%) | 166(100%) | 166(100%) | 166(100%) | 166(100%) | 166(100%) |
| 2 | 160(0%) | 160(0%) | 160(0%) | 160(0%) | 160(0%) | 160(0%) | 160(0%) |
| 3 | 194(0%) | 194(0%) | 141(0%) | 194(0%) | 12(0%) | 227(0%) | 194(0%) |

*S1.9. $NaFeO_2$*

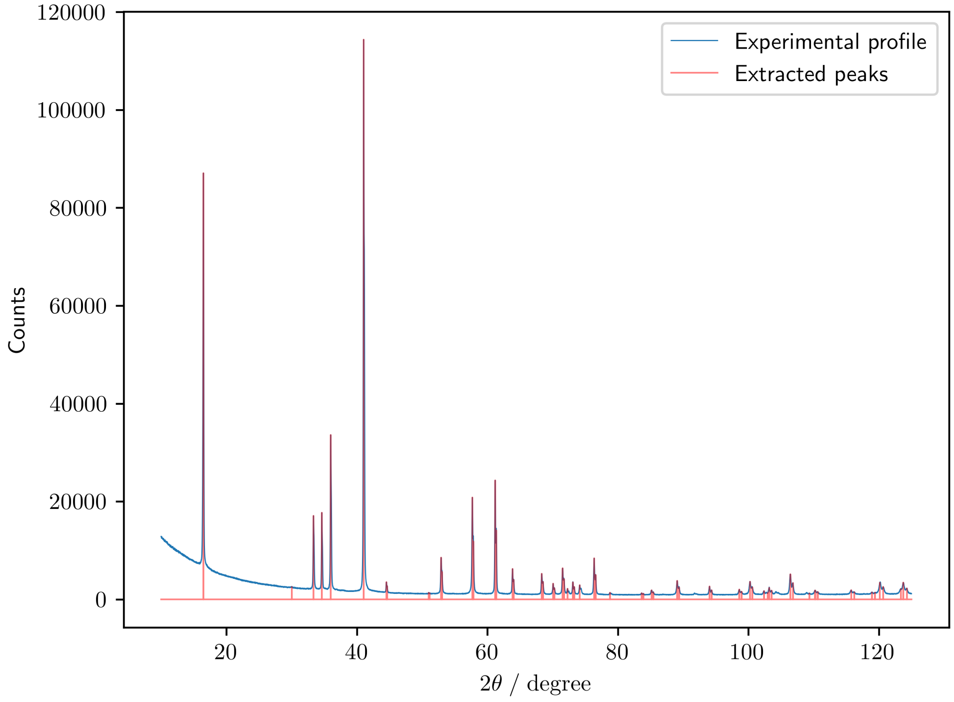


Fig. S9. Experimental diffraction profile of $NaFeO_2$ and the extracted peaks. The diffraction profile was measured with an X-ray diffractometer (RINT-TTRIII, Rigaku) using Cu-K$\alpha$ radiation (15 kV, 300 mA). The diffraction profile was recorded at a scanning rate of 2.0° / min in the $2\theta$ range of 10–125°.

Table SIX. *Space group (and its probability in parentheses) predicted by the six SpgVolNet models (*I–VI*) and their ensemble mean (EM) based on the peaks extracted from the experimental diffraction profile of $NaFeO_2$ shown in Fig. S9. The probability (0%) means it is below 0.5%. [Rietveld: $R\bar{3}m$ (166)]*

| | I | II | III | IV | V | VI | EM |
|---|---|---|---|---|---|---|---|
| 1 | 166(100%) | 166(100%) | 166(99%) | 166(99%) | 166(98%) | 166(100%) | 166(99%) |
| 2 | 160(0%) | 12(0%) | 12(1%) | 160(1%) | 12(2%) | 160(0%) | 12(0%) |
| 3 | 164(0%) | 164(0%) | 148(0%) | 164(1%) | 160(0%) | 12(0%) | 160(0%) |

**S2. Prediction of the simulated profile of $Li_2MnO_3$ under $C2/m$ and $R\bar{3}m$**

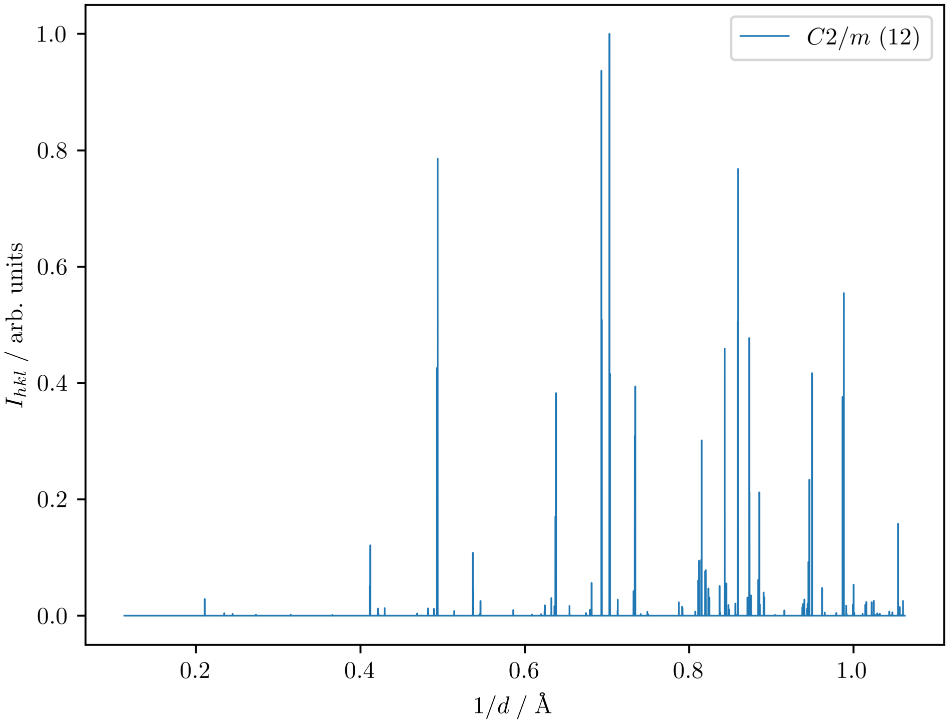


Fig. S10. The simulated profile of $Li_2MnO_3$ by constraining the Rietveld refinement under the space group to be $C2/m(12)$ for the SpgVolNet models.

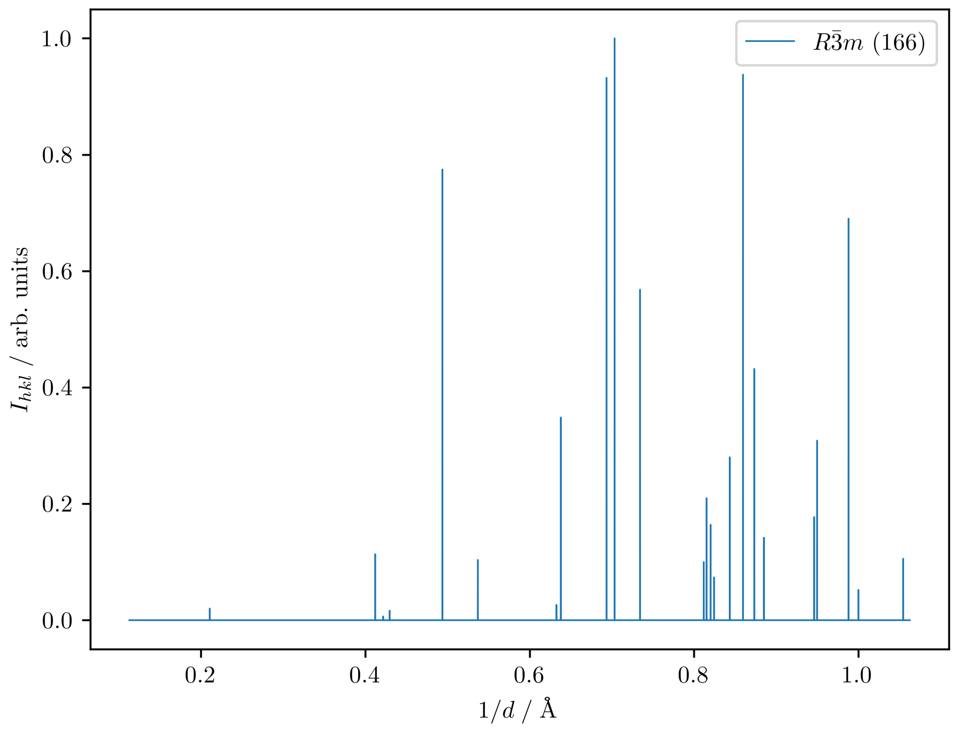


Fig. S11. The simulated profile of $Li_2MnO_3$ by constraining the Rietveld refinement under the space group to be $R\bar{3}m(166)$ for the SpgVolNet models.

Table SX. *Space group (and its probability in parentheses) predicted based on the ensemble mean probability of the six SpgVolNet models (*I–VI*) trained by five training datasets,* $D_i^{\mathrm{trn}}(\infty)$ $(1 \le i \le 5)$ *when the models were fed with the peak intensities and positions of the simulated profiles for* $Li_2MnO_3$ *shown in Figs. S10 and S11 by constraining the Rietveld refinement under the space group to be* $C2/m$*(12) and* $R\bar{3}m$*(166), respectively. The probability (0.0%) means it is below 0.05%.*

| | $D_1^{\mathrm{trn}}(\infty)$ | $D_2^{\mathrm{trn}}(\infty)$ | $D_3^{\mathrm{trn}}(\infty)$ | $D_4^{\mathrm{trn}}(\infty)$ | $D_5^{\mathrm{trn}}(\infty)$ |
|---|---|---|---|---|---|
| Rietveld refinement under $C2/m(12)$ | | | | | |
| 1 | 12(69.5%) | 12(77.7%) | 12(72.3%) | 12(79.0%) | 12(80.8%) |
| 2 | 166(6.9%) | 15(6.5%) | 166(8.1%) | 15(5.0%) | 15(7.5%) |
| 3 | 15(5.6%) | 166(4.2%) | 15(5.8%) | 2(3.7%) | 63(2.6%) |
| Rietveld refinement under $R\bar{3}m(166)$ | | | | | |
| 1 | 166(97.8%) | 166(99.9%) | 166(92.0%) | 166(99.9%) | 166(100.0%) |
| 2 | 227(2.1%) | 227(0.1%) | 227(7.9%) | 227(0.1%) | 227(0.0%) |
| 3 | 141(0.1%) | 141(0.0%) | 141(0.0%) | 160(0.0%) | 160(0.0%) |

## S3. An example of confusion matrix

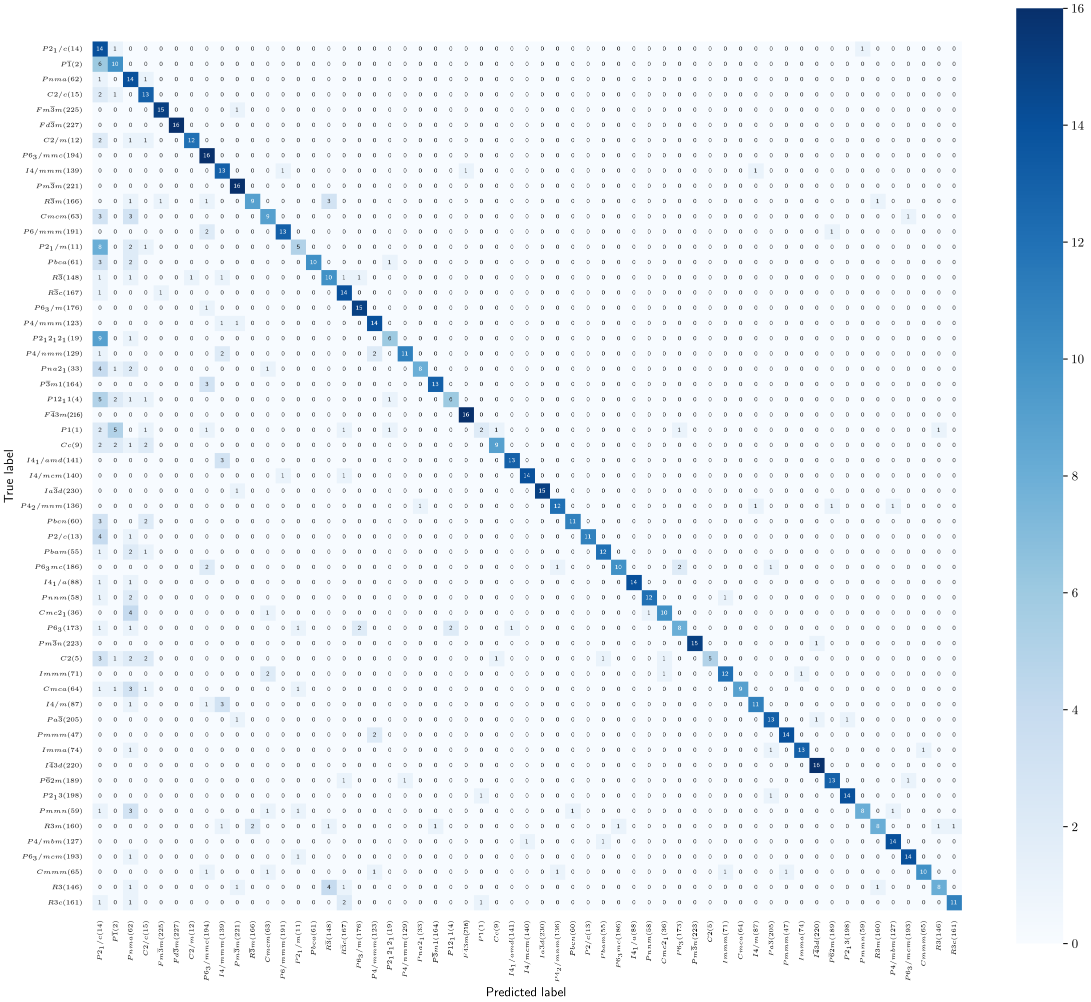


Fig. S12. An example of confusion matrix of space group classification on the SpgVol-Net model I with $D_1^{\mathrm{yld}}$. The true and predicted labels are ordered by the number of entries in the space group to which each belongs.

## S4. Effect of excluding outliers

Table SXI. *Effect of excluding outliers from the dataset on the statistical metrics of the SpgNet models.*

| | | Precision | Recall | F1 score |
|---|---|---|---|---|
| $D^{\rm trn}(\infty)$ | specific | 80.18±0.41 | 70.99±0.52 | 72.30±0.55 |
| | ensemble | 82.22±0.45 | 72.08±0.12 | 73.7±1.3 |
| $D^{\rm trn}(2\sigma_\eta)$ | specific | 80.11±0.41 | 71.14±0.45 | 72.38±0.53 |
| | ensemble | 82.1±1.0 | 72.4±1.1 | 73.9±1.4 |
| $D^{\rm trn}(2\sigma_U)$ | specific | 80.06±0.42 | 70.85±0.53 | 72.16±0.58 |
| | ensemble | 81.71±0.82 | 72.1±1.4 | 73.6±1.6 |
| $D^{\rm trn}(2\sigma_{U+\eta})$ | specific | 79.79±0.42 | 70.62±0.58 | 71.97±0.62 |
| | ensemble | 82.0±1.1 | 72.2±1.6 | 73.8±1.7 |
| $D^{\rm trn}(\sigma_\eta)$ | specific | 78.91±0.42 | 69.94±0.50 | 71.11±0.53 |
| | ensemble | 81.0±1.0 | 71.1±1.2 | 72.5±1.3 |
| $D^{\rm trn}(\sigma_U)$ | specific | 79.62±0.43 | 70.35±0.51 | 71.62±0.58 |
| | ensemble | 81.56±0.90 | 71.8±1.4 | 73.3±1.6 |
| $D^{\rm trn}(\sigma_{U+\eta})$ | specific | 78.53±0.45 | 69.40±0.48 | 70.54±0.54 |
| | ensemble | 80.61±0.41 | 70.6±1.2 | 72.0±1.3 |

The dataset $D(2\sigma_{U+\eta})$ *etc.* excludes from the parent dataset $D(\infty)$ the entries lying outside of twice the standard deviation ($\pm 2\sigma$) of the normal distribution with respect to both the logarithmic cell volume, $U = \log V$ and the logarithmic atomic density $\eta = \log(n_a/V)$, where $n_a$ is the number of atoms in the primitive cell of the volume $V$ (*cf,* Sec. 2.4). The labels "specific" and "ensemble" stand for the two kind of averaging described in Sec. 4.1.1.

### S5. Accidental overlap in diffraction binning

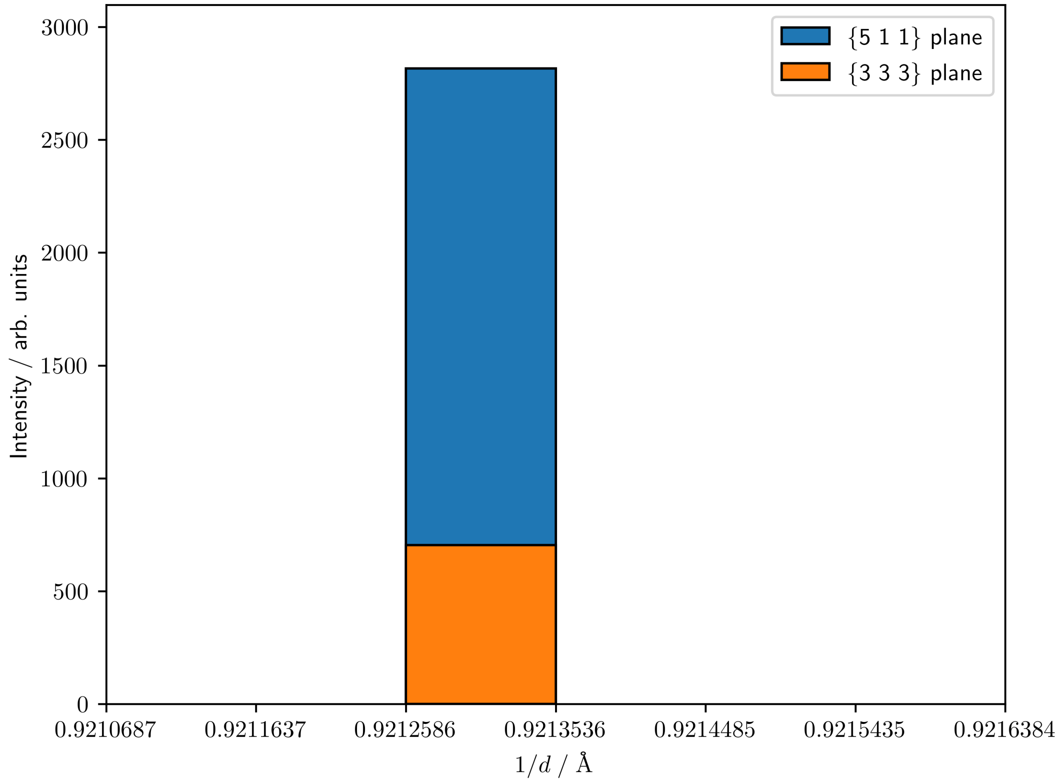


Fig. S13. An example of binning and accumulating the intensity $I_{hkl}$, where the diffraction from two distinct planes accidentally overlap and fall into an identical bin. NaCl: both planes, {511} and {333}, have $d^{-1}/\text{Å}^{-1} = 0.921304$.

## S6. Rietveld and Le Bail analysis of $Na_{2/3}(Mn_{2/3}Ni_{1/3})O_2$

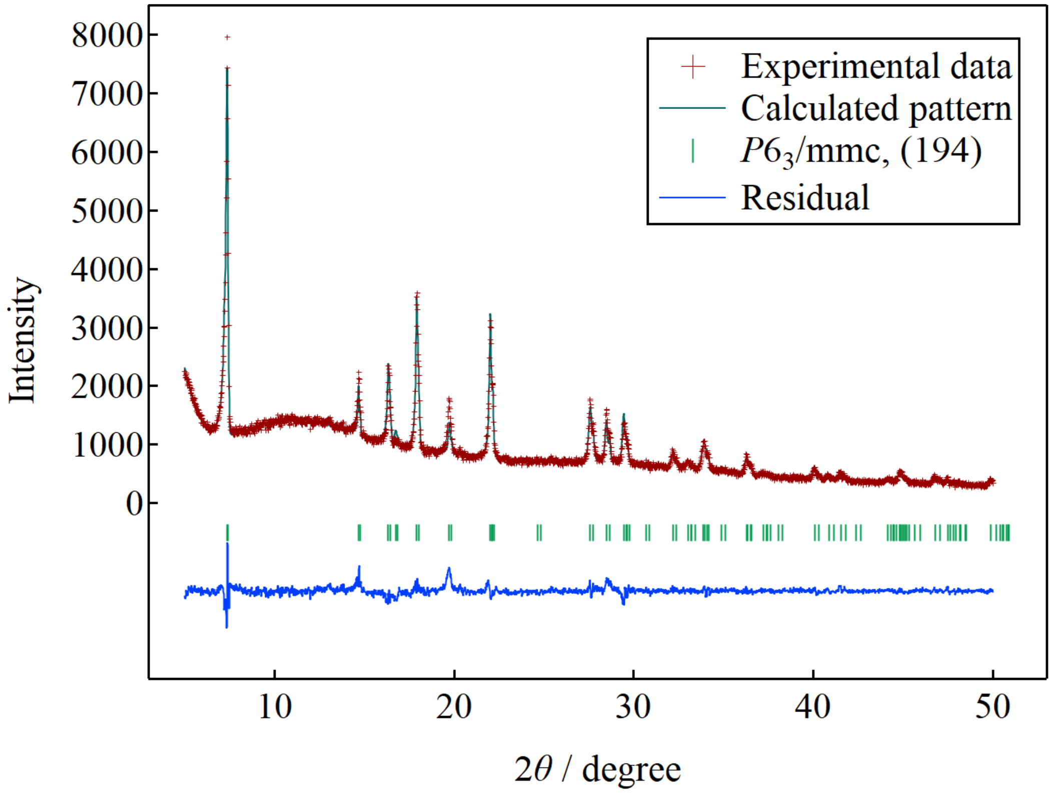


Fig. S14. Rietveld analysis of the experimental diffraction profile of $Na_{2/3}(Mn_{2/3}Ni_{1/3})O_2$ based on the space group $P6_3/mmc$. Reliability factors converged to $R_{\rm wp} = 5.76$ and $S \equiv R_{\rm wp}/R_{\rm e} = 1.71$, respectively.

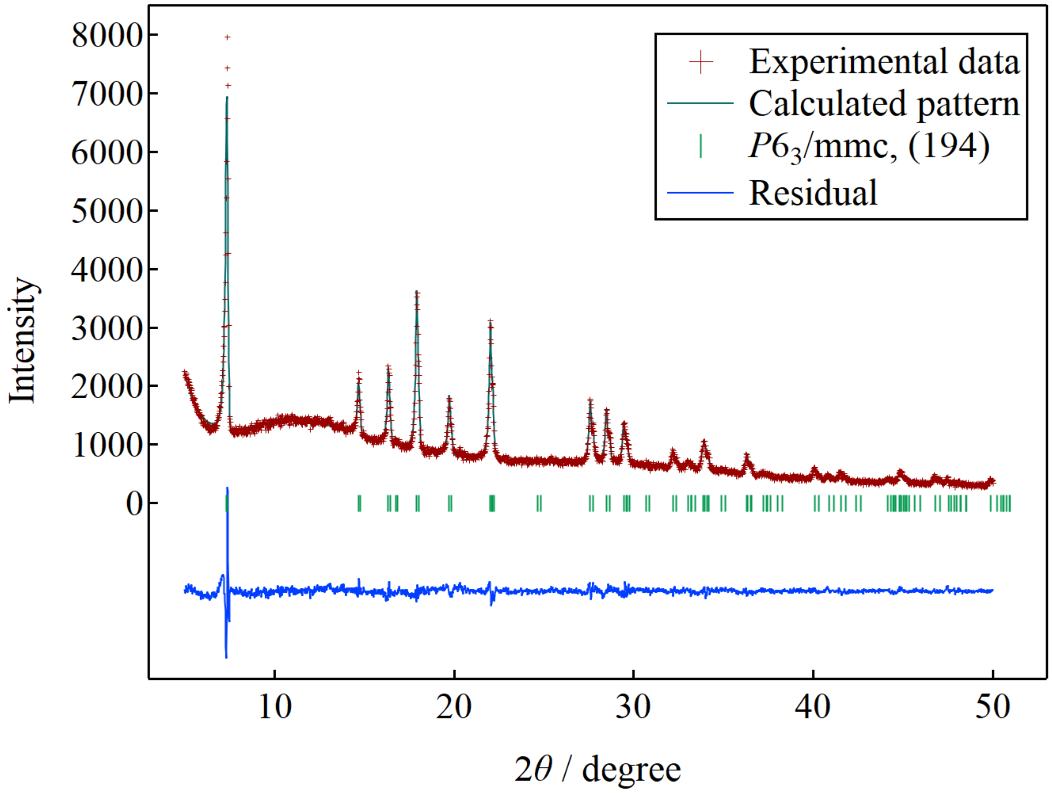


Fig. S15. Le Bail fit of the experimental diffraction profile of $Na_{2/3}(Mn_{2/3}Ni_{1/3})O_2$ based on the space group $P6_3/mmc$. Reliability factors converged to $R_{\rm wp} = 5.73$ and $S \equiv R_{\rm wp}/R_{\rm e} = 1.70$, respectively.
